# Identifying miRNA-mRNA Networks Associated With COPD Phenotypes

**DOI:** 10.3389/fgene.2021.748356

**Published:** 2021-10-28

**Authors:** Yonghua Zhuang, Brian D Hobbs, Craig P Hersh, Katerina Kechris

**Affiliations:** ^1^ Department of Biostatistics and Informatics, University of Colorado Anschutz Medical Campus, Aurora, CO, United States; ^2^ Channing Division of Network Medicine, Brigham and Women’s Hospital, Boston, MA, United States; ^3^ Division of Pulmonary and Critical Care Medicine, Brigham and Women’s Hospital, Boston, MA, United States; ^4^ Harvard Medical School, Boston, MA, United States

**Keywords:** microRNA, expression, chronic obstructive pulmonary disease, multi-omic, networks

## Abstract

Chronic obstructive pulmonary disease (COPD) is characterized by expiratory airflow limitation and symptoms such as shortness of breath. Although many studies have demonstrated dysregulated microRNA (miRNA) and gene (mRNA) expression in the pathogenesis of COPD, how miRNAs and mRNAs systematically interact and contribute to COPD development is still not clear. To gain a deeper understanding of the gene regulatory network underlying COPD pathogenesis, we used Sparse Multiple Canonical Correlation Network (SmCCNet) to integrate whole blood miRNA and RNA-sequencing data from 404 participants in the COPDGene study to identify novel miRNA–mRNA networks associated with COPD-related phenotypes including lung function and emphysema. We hypothesized that phenotype-directed interpretable miRNA–mRNA networks from SmCCNet would assist in the discovery of novel biomarkers that traditional single biomarker discovery methods (such as differential expression) might fail to discover. Additionally, we investigated whether adjusting -omics and clinical phenotypes data for covariates prior to integration would increase the statistical power for network identification. Our study demonstrated that partial covariate adjustment for age, sex, race, and CT scanner model (in the quantitative emphysema networks) improved network identification when compared with no covariate adjustment. However, further adjustment for current smoking status and relative white blood cell (WBC) proportions sometimes weakened the power for identifying lung function and emphysema networks, a phenomenon which may be due to the correlation of smoking status and WBC counts with the COPD-related phenotypes. With partial covariate adjustment, we found six miRNA–mRNA networks associated with COPD-related phenotypes. One network consists of 2 miRNAs and 28 mRNAs which had a 0.33 correlation (*p* = 5.40E-12) to forced expiratory volume in 1 s (FEV_1_) percent predicted. We also found a network of 5 miRNAs and 81 mRNAs that had a 0.45 correlation (*p* = 8.80E-22) to percent emphysema. The miRNA–mRNA networks associated with COPD traits provide a systems view of COPD pathogenesis and complements biomarker identification with individual miRNA or mRNA expression data.

## Introduction

Chronic obstructive pulmonary disease (COPD) is the third leading cause of death worldwide (Celli and Wedzicha, 2019) and is primarily attributable to the effects of cigarette smoking. Although smoke exposure drives COPD, we still have a poor understanding of the molecular traits and biologic pathways that are associated with specific COPD-related traits (Carolan et al., 2014). In addition, patients with COPD exhibit heterogeneity in clinical presentation, with different morbidities and prognoses for each phenotype (Bowler et al., 2015). Different COPD-related phenotypes might be attributable to different molecular mechanisms, such as miRNA–mRNA networks.

MicroRNAs (miRNAs) are a type of small non-coding RNAs that are approximately 21–25 nucleotides long and play important roles in regulating both gene and protein levels by binding to mRNAs to contribute to either transcript degradation or inhibition of protein translation. A single miRNA may regulate tens to hundreds of genes simultaneously due to the redundancy of complementary sequences between miRNAs and target sequences in the 3′UTR of mRNA(s). Many studies have implicated miRNAs in the pathogenesis of COPD (Osei et al., 2015; Salimian et al., 2018; Keller et al., 2019).

Although some COPD biomarker investigations have focused on single candidate mRNA or miRNA (e.g., IRF-3, miR-199a-5p) (Ishii et al., 2017; Takei et al., 2019), there is no single mRNA, miRNA, or other molecule that can fully explain the development of COPD. Compared with single biomarkers, panels of several biomarkers have been shown to improve predictive accuracy of disease severity, progression, and mortality in COPD (Zemans et al., 2017). Furthermore, a network-based approach could further pinpoint potential mechanisms by integrating datasets and thereby increasing statistical power. This approach has been used to integrate proteins and metabolomics to study their combined relationship to COPD (Mastej et al., 2020).

Networks are a natural framework to represent relationships between molecular components (Winterbach et al., 2013). A network consists of a series of nodes, or biological entities such as miRNAs and genes. The direct and indirect interactions between miRNAs and mRNAs form regulatory network(s) that contribute to biological processes. Networks provide a graphical representation of molecular interactions that may explain pathogenesis for complex diseases (Civelek and Lusis, 2014).

For this study, we used our recently developed tool called sparse multiple canonical correlation network (SmCCNet) (Shi et al., 2019) to integrate blood-based miRNA and mRNA data into COPD-associated miRNA–mRNA gene regulatory networks. SmCCNet uses a canonical correlation-based approach to simultaneously integrate multi-omics data and a quantitative phenotype of interest to build interpretable networks.

Unlike standard pairwise correlations between individual features, canonical correlation measures the relatedness of two sets of features simultaneously by finding a linear combination of members from each set. SmCCNet is an extension of canonical correlation in which linear combinations are found to maximize the correlation between multi-omics datasets (e.g., miRNAs, mRNAs) and a phenotype of interest (e.g., FEV_1_pp or emphysema). We have previously published the details of the SmCCNet method (Shi et al., 2019), where we inferred miRNA–mRNA networks associated with COPD phenotypes in a pilot study of 27 subjects. In the current study, we further used SmCCNet to integrate whole blood miRNA and mRNA expression data from 404 participants from the COPDGene Study, which is one of the most comprehensive sets of blood miRNA and mRNA data available to date for COPD to identify novel miRNA–mRNA networks associated with lung function and emphysema. Our aim was to integrate -omic data to build interpretable networks that could assist in the discovery of novel biomarkers that might have been overlooked in standard biomarker discovery methods.

## Materials and Methods

### Transcriptomics Data (mRNA and miRNA Seq Data) and Data Processing

These data were generated as part of the COPDGene Study, which is one of the largest studies ever that enrolled 10,198 participants with and without COPD between 2007 and 2011 (Visit 1, phase I study) to identify genetic factors associated with COPD (Ragland et al., 2019; Regan et al., 2019). The miRNA and mRNA high-throughput sequencing data were from peripheral blood samples collected at the 5-years follow up visits from 2013 to 2017 (phase II study).

**FIGURE 1:**
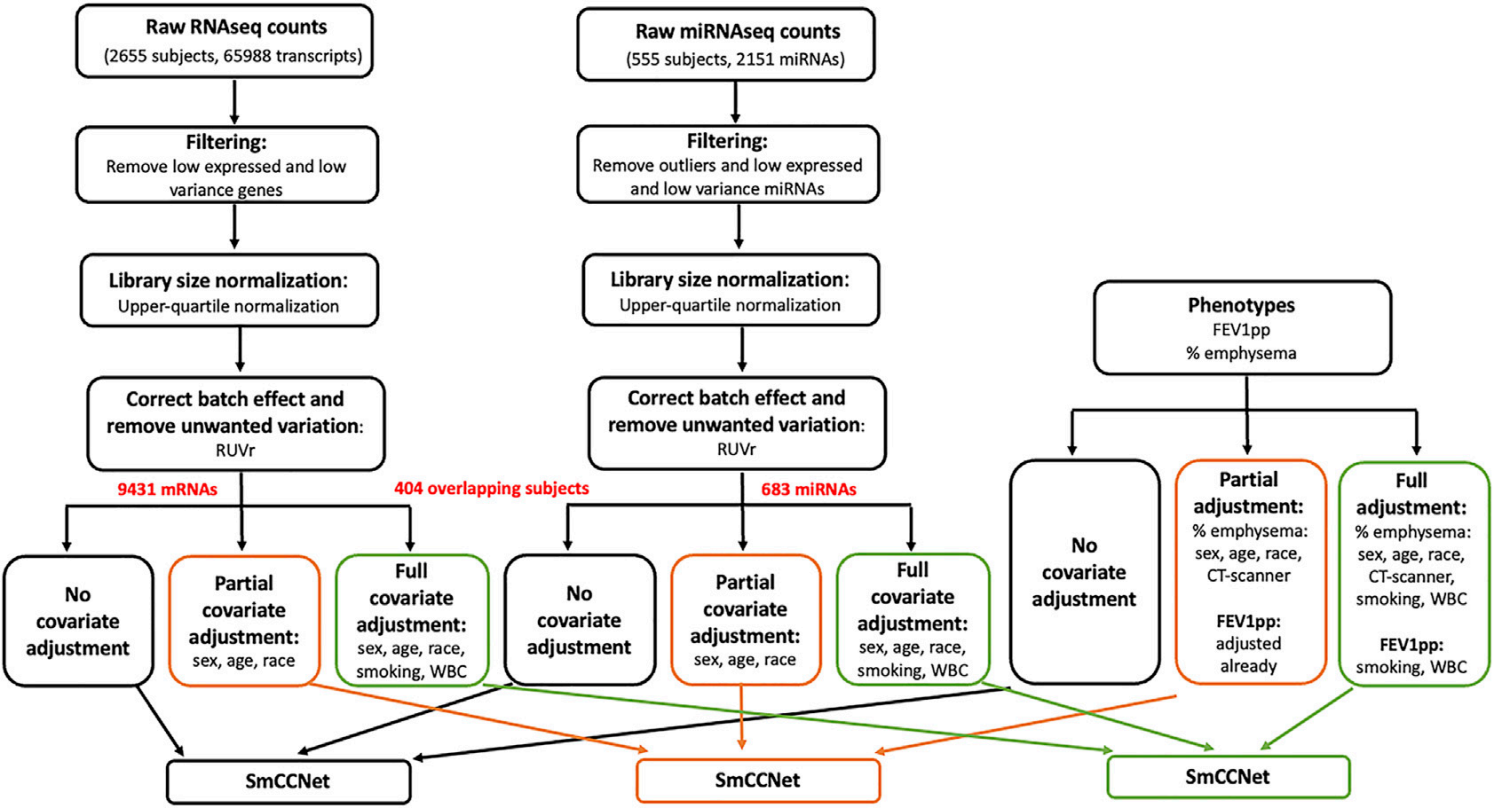
Flowchart of data preprocessing and covariate adjustment for SmCCNet analysis. The miRNA (*n* = 555) and mRNA (*n* = 2655) high-throughput sequencing data were from peripheral blood samples collected at COPDgene phase II study. For RNAseq gene count data, the low-expressed and low-variance genes were removed. Specifically, we first filtered down to protein coding genes. Then for each gene, we required more than 10 reads in at least 500 samples, and the standard deviation to be greater than 30. The upper-quartile normalization and RUVr (remove unwanted variation using residuals) methods were applied to normalize the library size, correct batch effect and remove unwanted variation. The resultant data were either preserved or further adjusted with two different sets of covariates [partial adjustment: sex, age and race; full adjustment: sex, age, race, current smoking status and white blood cell (WBC) percentages]. The miRNA expression count was processed similarly to the gene count data; 151 samples of miRNA data were removed due to low sequencing depth, missing clinical phenotypes, or being in a bad batch (see detail in Methods). After removing three outlier miRNAs with large counts (individual reads >5 million), we filtered out “absent” and low-variant miRNAs by requiring more than 10 reads in at least 200 subjects, as well as a minimum standard deviation of 10 across subjects. The clinical phenotypes including FEV_1_pp and % emphysema were processed and adjusted correspondingly to match mRNA and miRNA data (*n* = 404) for the downstream SmCCNet analysis. Of note, FEV_1_pp is already adjusted for sex, age, and race. Thus, no additional adjustment was needed in the partial adjustment. In addition, CT scanner model is a potential cofounder for percent emphysema, and it was included in the partial and full adjustment for percent emphysema.

The total miRNA dataset consisted of 555 peripheral blood samples and 2151 miRNAs (LaBelle et al., 2021). In a pilot study, we observed abundant hemolysis-related miRNAs including hsa-miR-486-5p, hsa-miR-451a, and hsa-miR-92a-3p in RNA seq data. Therefore, we used single multiplexed blocking oligonucleotides to reduce these unwanted hemolysis-related miRNAs before sequencing RNA and allow for better detection of lowly expressed biomarkers (LaBelle et al., 2021). We removed 15 samples due to either low sequencing depth (total read counts <200 k) or missing clinical information. In addition, the plate5 batch in the microRNA data appeared to be fundamentally different than all other batches, and the samples in plate5 were removed; 404 subjects remained for further preprocessing. We first removed three outlier miRNAs with large counts (individual reads >5 million) including hsa-miR-191-5p, hsa-miR-486-5p and hsa-miR-92a-3p. Of note, hsa-miR-486-5p and hsa-miR-92a-3p are hemolysis-susceptible miRNAs (Kirschner et al., 2013). Although the majority of hsa-miR-486-5p and hsa-miR-92a-3p were removed with blocking oligonucleotides, their remaining expression values were still relatively high. We also filtered out “absent” and low-variant miRNAs by requiring more than 10 reads in at least 200 subjects, as well as a minimum standard deviation of 10 across subjects (Conesa et al., 2016). The number of miRNAs reduced to 683 post-filtering. To normalize the miRNA expression data, we applied upper-quartile normalization and Remove Unwanted Variations with Residuals (RUVr) (Risso et al., 2014). The generalized linear model used in RUVr to determine residuals includes the following covariates: sex, race, age, smoking status, white blood cell percentages from the complete blood count (CBC, including neutrophils, lymphocytes, eosinophils), and forced expiratory volume during the first second expressed as a percent of predicted value (FEV_1_pp). Finally, the corrected sequencing counts were transformed to be homoscedastic via a variance stabilizing transformation (VST) (Anders and Huber, 2010) (Figure 1).

The full CODPGene total mRNA sequencing data contained 2655 peripheral blood samples on 65,988 transcripts. For the genes, we first filtered down to protein coding genes (21,835/65,988). Then for each gene, we required more than 10 reads in at least 500 samples, and the standard deviation to be greater than 30. Post filtering, we obtained 2655 samples and 9,430 genes. As with the miRNA data, we applied upper-quartile normalization and RUVr (Risso et al., 2014) with the same generalized linear model and covariates to remove unwanted variance including batch effect. Finally, the corrected sequencing counts were transformed to be homoscedastic via VST (Anders and Huber, 2010).

For the mRNA sequencing data, 555 subjects were also profiled in the miRNA sequencing study. As described above, we removed 151 samples of miRNAs data due to either low sequencing depth, missing clinical phenotypes or in a bad batch. We extracted the preprocessed mRNA data to match the preprocessed miRNAs on the same 404 subjects with phenotypes (Figure 1). The network analysis with SmCCNet was performed on the 404 paired samples only, which included 183 controls, 169 COPD cases, and 52 subjects with Preserved Ratio Impaired Spirometry (PRISm).

### Clinical Variables and Definitions

We focused on two COPD phenotypes: percent predicted forced expiratory volume in one second (FEV_1_pp) and percent emphysema. A measure of lung function, FEV_1_pp is the amount of air one can forcibly exhale in one second (L) divided by the predicted FEV_1_ adjusted for age, height, race, and sex (Hankinson et al., 1999). Emphysema, a destruction of distal airspaces, is associated with the clinical severity of COPD (Li et al., 2019) but is loosely correlated with FEV_1_pp. Percent emphysema is an imaging phenotype defined as percent of lung voxels with attenuation values less than −950 Hounsfield Units on quantitative analysis of chest computed tomography (CT) scans.

Individuals were classified as having normal spirometry if FEV_1_ ≥80% and FEV_1_/FVC >0.7. In participants with COPD (FEV_1_/FVC <0.7), the Global Obstructive Lung disease (GOLD) system was used to grade the severity of airflow limitation: early, GOLD 1 (FEV_1_ ≥80%); moderate, GOLD 2 (50% ≤ FEV_1_ < 80%); severe, GOLD 3 (30% ≤ FEV_1_ < 50%); and very severe, GOLD 4 (FEV_1_ <30%). Individuals with FEV_1_ <80% but FEV_1_/FVC >0.7 were classified as Preserved Ratio Impaired Spirometry (PRISm) (Wan et al., 2014). FEV_1_pp and percent emphysema variables were both centered and scaled.

### Covariate-Adjusted Omics Data

Although covariates may influence miRNA and mRNA abundance in human blood studies, covariate adjustment is often missing in many -omic studies, especially network analysis. There is no consensus on the effect covariate adjustment has on data and many covariates can also be associated with disease variables. In addition, it is not clear if the confounder effects for epidemiologic studies and single omics analyses are translatable to network integration studies. To investigate the effect of covariate adjustments for network analysis, we prepared the data in three ways: 1) no covariate adjustment; 2) partial covariate adjustment; 3) full covariate adjustment (Figure 1). Of note, the COPDgene study has collected 905 clinical variables. In this study, we selected a small subset of potential covariates based on co-author clinical expertise and previous literature (Coxson et al., 2013; Bradford et al., 2017; Willinger et al., 2017).

In partial covariate adjustment, we adjusted the mRNA and miRNAs data for sex, age, and race. Regarding the phenotypes for the partial covariate adjustment, since FEV_1_pp has been already adjusted for sex, age, and race, no further adjustment for the phenotype FEV_1_pp is required in partial covariate adjustment. For percent emphysema, we adjusted for sex, age race, and CT scanner model, since the latter is a potential confounder for percent emphysema.

In full covariate adjustment, we adjusted the mRNA and miRNAs data for sex, age, race, smoking, and white blood cell (WBC) percentages including percent lymphocytes, neutrophils, and eosinophils. Regarding the phenotypes for full covariate adjustment, FEV_1_pp was further adjusted for current smoking and WBC. For percent emphysema, we adjusted for sex, age race, CT scanner model, current smoking, and WBC percentages. Covariate adjustment was performed using linear regression for each mRNA/miRNA/phenotype with the listed covariates as the predictors. Residuals from these models were utilized in adjusted models moving forward.

### Sparse Multiple Canonical Correlation Network

miRNA–mRNA networks correlated to FEV_1_pp and percent emphysema were constructed using SmCCNet (Figure 1), a technique developed previously in our group (Shi et al., 2019) that uses multiple canonical correlation network analysis to integrate multi-omics data types with a phenotype of interest.

Before applying SmCCNet, the Pearson correlation matrices were calculated between the -omics data (X_1_ for mRNA, X_2_ for miRNA) and the phenotype of interest (*Y*). The range of correlations of between the -omic data was comparable to the range of correlations of mRNA-omic data and the phenotype of interest. However, the range of correlations between the miRNA-omic data and the phenotype of interest was weaker. Thus, we applied a scaled version of SmCCNet (i.e., *a*, *b*, and *c* are not all equal) to prioritize the correlations between the miRNA-omic data and the phenotype of interest. The scaling factors and sparse penalty parameters (l_1_, l_2_) were chosen through a fourfold cross validation in a grid search (See details in Supplementary method). Besides K-fold cross validation, the SmCCNet framework also provides feature subsampling to create robust network construction (Shi et al., 2019). Since the number of miRNAs is much smaller, we chose the subsampling proportions to be 70% and 90% for mRNA and miRNA, respectively. The subsampling procedure was repeated 1,000 times in this study.

### Edge Thresholds

Lastly, after miRNA–mRNA networks were generated from SmCCNet, absolute edge thresholds were applied to the networks to filter out weak edges (edges with low weights) (Shi et al., 2019). Edge thresholds were systematically testing starting at 0.001, to the maximum of adjacency matrix in each module, in increments of 0.001 to reveal trimmed, interpretable networks with strong edges that still had strong correlations to the phenotype of interest and a balanced miRNA to mRNA ratio.

### miRNA–mRNA Network Correlations With Phenotypes

To determine the quality of each network, we calculated the Pearson correlation of the first principal component (PC1) of the network and the phenotype of interest. PC1 was selected as a single summary of the network because it explains the most variance in the expression data of the nodes in the network. Identified FEV_1_pp and percent emphysema associated networks were visualized using Cytoscape version 3.8 (Shannon et al., 2003).

### COPD-Associated Network Quality Assessment

To assess the effects of covariate adjustment networks that were constructed on unadjusted, partially-adjusted and fully-adjusted -omic data and to compare the quality of COPD-associated networks, the pairs of miRNA and mRNA with negative correlation in the identified network were queried in multiple microRNAs/targets databases, including validated microRNA-target databases (miRecords, miRTarBase, and TarBase), predicted microRNA-target databases (DIANA-microT, ElMMo, MicroCosm, miRanda, miRDB, PicTar, PITA, and TargetScan), and microRNA-disease/drugdatabases (miR2disease, Pharmaco-miR VerSe, and PhenomiR). Of note, the correlation direction was determined by calculating Pearson’s correlation for each pair of mRNA and miRNA based on their expression data. The queries were performed with the “multiMiR” R package (Ru et al., 2014).

Since the microRNA-target databases are not COPD-specific, we further compared the miRNAs in the identified networks with the published miRNAs associated with COPD phenotypes. In the review paper of Osei et al. (2015), 70 unique miRNAs related to “FEV1,” “emphysema,” or other COPD relevant phenotypes were summarized. In the recent review papers of Salimian et al. (2018) and Dutta et al. (2019), 174 miRNAs associated with COPD were summarized. The COPD-associated miRNAs were combined in these three reviews. For some COPD-associated miRNAs, 3′ or 5′ information was not reported in the original research studies. Therefore, we used both 3′ and 5′ for those miRNAs. The final set was 289 COPD-related miRNAs for comparison with miRNAs in our results.

### Gene Ontology Enrichment Analysis on Chronic Obstructive Pulmonary Disease-Associated Network

We performed gene ontology (GO) enrichment analysis with Fisher’s exact test on the gene nodes in the COPD-associated networks to identify important pathways related to COPD phenotypes. Gene ontology provides a controlled vocabulary for describing biological processes (BP), molecular functions (MF) and cellular components (CC). We focused on BP ontology enrichment analysis since we are interested in what biological processes are involved in COPD. The GO size parameter was set to be 30 to prune the GO hierarchy from the terms which have less than 30 annotated genes. Besides running classic GO enrichment tests, we also took into account GO hierarchy into account and performed conditional enrichment analysis with the “weight01” algorithm in “topGO” R package (Alexa et al., 2006). Under GO hierarchy, one parent GO gene set is likely to be enriched when one of its offspring gene set is enriched. Conditional enrichment analysis accounts for this phenomenon in analyzing enrichment significance. In conditional enrichment analysis, all leaf gene sets are tested as conventional enrichment analysis. Then, parents of these gene sets are examined as follows: if one of their offspring is significant, the genes belonging to this child are removed from the parent gene set. The analysis is performed recursively until reaching the root gene set.

### Single Omics Analysis for Comparison

We performed Pearson correlation analysis on the mRNAs or miRNAs and COPD phenotypes including FEV_1_pp and percent emphysema. The significant genes and miRNAs were selected for comparison if adjusted *p*-values <0.05 after Benjamini-Hochberg multiple testing corrections.

### Statistical Package

All analyses were performed using the statistical R v4.0 software. The following R packages: “SmCCNet v0.99” (Shi et al., 2019), “WGCNA v1.66 (Langfelder and Horvath, 2008),” “topGO v2.44 (Alexa et al., 2006),” and “multiMiR v1.14 (Ru et al., 2014)” were used for gene network analysis.

## Results

### Clinical Characteristics of Subjects

The samples in this miRNA–mRNA network study covered a range of spirometry profiles including normal (183), COPD with all four grades of GOLD airflow limitation severity (GOLD 1: 47; GOLD 2: 68; GOLD 3: 37; GOLD 4: 17), and Preserved Ratio Impaired Spirometry category (PRISm: 52). We categorized the samples into three groups: normal spirometry, COPD cases (GOLD = 1–4) and PRISm (Table 1). There are differences in age, BMI, neutrophil percent, lymphocyte percent, FEV_1_pp, FVCpp and percent emphysema in the three different groups (*p* < 0.05). However, sex and race are not statistically different (*p* > 0.05).

**TABLE 1:** Clinical characteristics of overlapping mRNA and miRNA dataset.

Clinical Variables	Normal Spirometry (n = 183)	COPD (GOLD 1–4) (n = 169)	PRISm (n = 52)	*p*-Values
Sex, Male (%)	79 (43.2)	84 (49.7)	24 (46.2)	0.47
Race, Non-Hispanic White (%)	134 (73.2)	128 (75.7)	36 (69.2)	0.631
Race, African Americans (%)	49 (26.8)	93 (24.3)	16 (30.8)	
Age (y)	62.98 (8.20)	68.28 (8.67)	61.04 (7.02)	<0.001
BMI (kg/m^2^)	28.92 (5.84)	28.18 (6.09)	32.34 (6.15)	<0.001
Current smoking (%)	60 (32.8)	64 (37.9)	27 (51.9)	0.041
Percent of neutrophil	57.28 (10.76)	61.62 (10.64)	59.00 (7.64)	0.001
Percent of lymphocyte	31.81 (10.22)	26.51 (9.24)	29.98 (7.75)	<0.001
Percent of eosinophil	2.62 (1.98)	2.69 (1.98)	2.56 (1.65)	0.903
FEV_1_% predicted (FEV_1_pp)	97.62 (12.40)	63.75 (23.76)	66.87 (9.61)	<0.001
FVC % predicted (FVCpp)	95.26 (11.84)	84.85 (20.25)	67.38 (10.38)	<0.001
Percent emphysema	1.61 (2.39)	9.50 (11.45)	0.96 (1.88)	<0.001

Data are presented as the mean (standard deviation) for age, body mass index (BMI), FEV_1_% predicted, percent neutrophil, percent lymphocyte, percent eosinophil, and percent emphysema. COPD: chronic obstructive pulmonary disease. PRISm: Preserved Ratio Impaired Spirometry defines individuals with a reduced FEV_1_ but with a preserved FEV_1_/FVC where FVC is forced vital capacity. GOLD: the Global Obstructive Lung Disease system for grading COPD severity: GOLD 1 is early COPD, GOLD 2 is moderate COPD, GOLD 3 is severe COPD, GOLD 4 is very severe COPD, and GOLD 0 is an individual without COPD (control). FEV_1_%: percent predicted forced expiratory volume in one second. Percent emphysema: percent of lung voxels less than −950 Hounsfield Units on inspiratory CT scans.

### Correlations Between Adjusted -Omic Data and Phenotype

**FIGURE 2:**
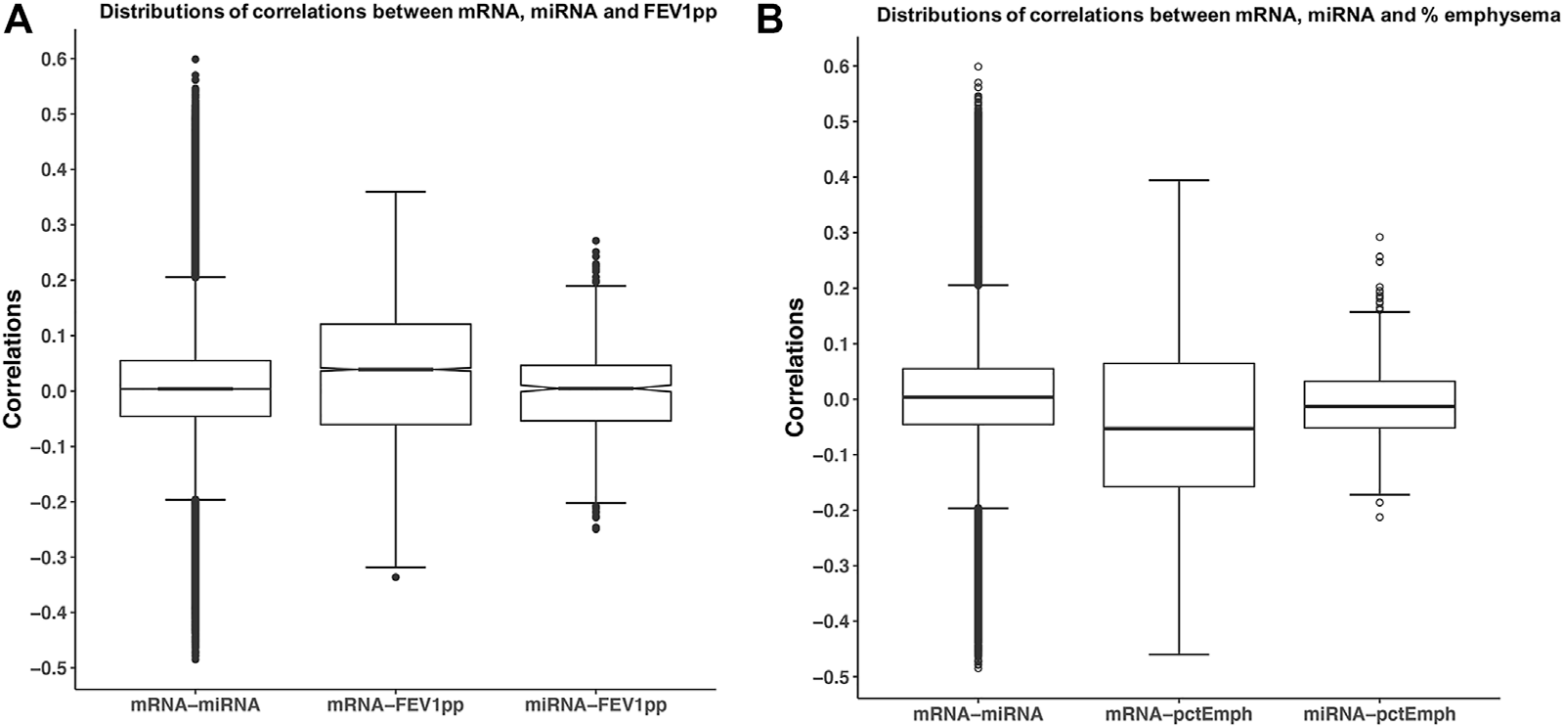
Correlations of mRNA, miRNA, and phenotypes. The miRNA and mRNA data were partially adjusted as discussed in method session. Each pair of correlations among mRNA and miRNA, mRNA, and phenotype. **(A)** FEV_1_pp or **(B)** percent emphysema was calculated. Boxplots illustrate the distribution of pair-wise correlations.

Before applying SmCCNet to -omic data, we explored the range of correlations between -omic datasets and between-omic data and the phenotype of interest. The range of correlations between the partial adjusted mRNA data and the adjusted miRNA data was −0.48 to 0.60. The range of correlations between the adjusted mRNA and the FEV_1_pp was −0.34 to 0.36, but the correlations between the adjusted miRNA and the FEV_1_pp was smaller and in the −0.25 and 0.27 range (Figure 2A). The range of correlations between the adjusted mRNA and the percent emphysema was −0.46 to 0.40, but the correlations between the adjusted miRNA and the percent emphysema was smaller and in the −0.21 and 0.29 range (Figure 2B). Of note, the similar patterns of pair correlation distributions were observed in the unadjusted and fully adjusted datasets (data not shown).

The range of correlations between the adjusted miRNA and phenotype data was smaller than the range of correlations of mRNA–miRNA and mRNA–phenotypes likely because the total number of miRNAs is smaller. However, this discrepancy can result in networks that are driven by mRNA related correlations and ignore potentially important correlations between miRNA and the phenotype(s). Therefore, additional emphasis was made on the correlations between miRNAs data and the phenotypes with the scaled version of SmCCNet applied to the miRNA and mRNA data as discussed in the Methods section.

The final scaling factors for SmCCNet were selected through a four-fold cross validation to find the pair that minimized the prediction error (Supplementary Table S1).

### Identified COPD Networks with Different Covariate Adjustments

Scaled SmCCNet was applied on the unadjusted, partially-adjusted, or fully-adjusted miRNA and mRNA data. As summarized in Table 2 and Supplementary Figure S1, we identified 4, 11, and 7 FEV_1_pp-associated modules in unadjusted, partially-adjusted and fully-adjusted data, respectively. The number of miRNAs in the identified modules ranges from 1 to 6. The median ratio of the number of miRNAs to mRNA was 0.01 (unadjusted and fully-adjusted) or 0.02 (partially-adjusted).

**TABLE 2:** Summary of FEV_1_pp-related modules.

Covariate adjustments	Unadjusted	Partial	Full
**n**	4	11	7
No. miRNA [median (min, max)]	2.00 (1.00, 5.00)	1.00 (1.00, 6.00)	2.00 (1.00, 5.00)
No. mRNA [median (min, max)]	328.50 (82.00, 884.00)	61.00 (7.00, 1,003.00)	71.00 (16.00, 1,364.00)
Ratio [median (min, max)]	0.01 (0.00, 0.01)	0.02 (0.00, 0.14)	0.01 (0.00, 0.09)
propVar [median (min, max)]	0.45 (0.42, 0.60)	0.40 (0.16, 0.56)	0.23 (0.13, 0.30)

Notes: propVar, percent variance explained; No., number; min, minimum; max, maximum.

The emphysema-associated modules derived from unadjusted, partially-adjusted and fully-adjusted data are summarized in Supplementary Figure S2 and Supplementary Table S2. We identified 4, 7, and 17 emphysema-associated raw modules in unadjusted, partially-adjusted and fully-adjusted data, respectively. The number of miRNAs in the identified modules ranges from 1 to 96. The median ratio of the number of miRNA and mRNA is 0.08 (unadjusted), 0.01 (partially-adjusted) and 0.14 (fully-adjusted), respectively.

### Comparison of COPD Networks with Different Covariate Adjustments

**FIGURE 3:**
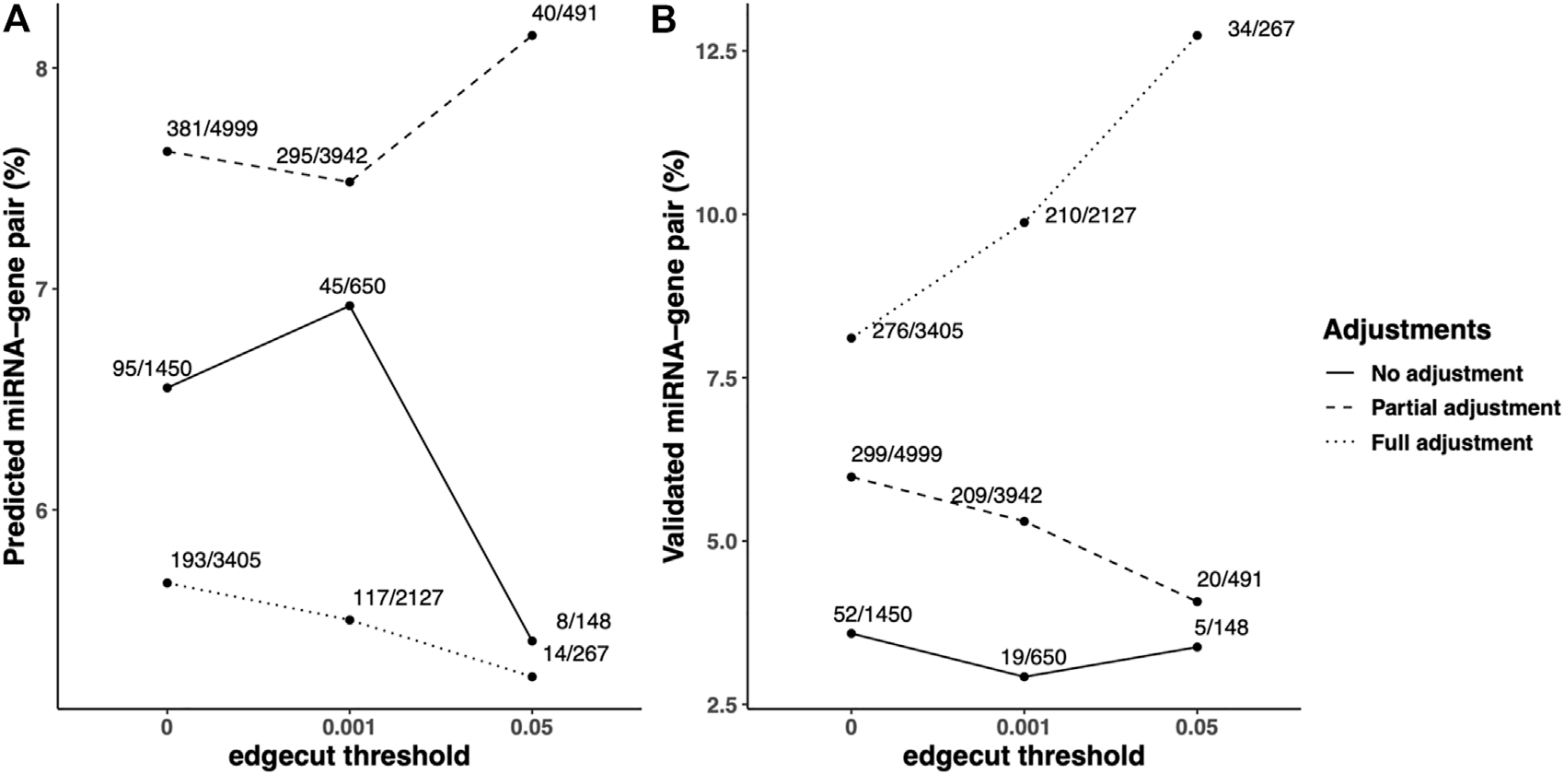
Predicted/validated miRNA–mRNA pairs percentage in the identified FEV_1_pp-networks with different adjustment strategies. Scaled SmCCNet were applied on the unadjusted, partially adjusted or fully adjusted miRNA and mRNA data with the FEV_1_pp phenotype. To compare the quality of FEV_1_pp-associated networks, the pairs of miRNA and mRNA (gene) with negative correlation in the identified networks were queried in multiple microRNA/target databases, including predicted microRNA-target databases and validated microRNA-target databases. The queries were performed with the “multiMiR” R package as discussed in the Methods. **(A and B)** The ratio of predicted **(A)** or validated **(B)** pairs in databases and total pairs in the constructed networks. We applied 0.001 and 0.05 edge thresholds to filter weak edges between miRNAs and mRNAs, the ratio of predicted miRNA–mRNA in unadjusted, partially adjusted, and fully adjustment resulted networks were updated correspondingly.

To compare the quality of the COPD-associated networks, the pairs of miRNA and mRNA (gene) with negative correlation in the identified networks were queried in multiple microRNA/target databases, including predicted microRNA-target databases and validated microRNA-target databases. In the identified FEV_1_pp networks, we found 1,450, 4,999, and 3405 miRNA–mRNA pairs in unadjusted, partially-adjusted and fully-adjusted data (Figure 3). Among these identified pairs, 95, 381, and 193 were found in the predicted miRNA–mRNA databases, which are determined based on sequence information of potential targets. The percentage of predicted miRNA–mRNA amongst all miRNA–mRNA edges in the network was highest in the partial adjusted networks (7.62%, Figure 3A). The partial adjusted networks also had the highest percentage of predicted miRNA–mRNA when we applied the 0.001 and 0.05 edge threshold (7.48% and 8.15% respectively, Figure 3A). In addition, we found 52, 299 and 276 pairs of identified miRNA and mRNA have been validated in the miRNA–mRNA databases. The highest percentage of validated miRNA–mRNA amongst all miRNA–mRNA edges in the network was for the fully-adjusted network (Figure 3B) regardless of edge threshold, but the partially adjusted network had the next highest percentages. In summary, compared with unadjusted control, the partial adjustment had higher percentages of accuracies of miRNA–mRNA pairs when mapping with predicted and validated databases. Although the full adjustment had higher percentages of accuracies of miRNA–mRNA pairs when mapping with validated databases, it had the lowest percentage of accuracies of predicted miRNA–mRNA pairs.

**FIGURE 4:**
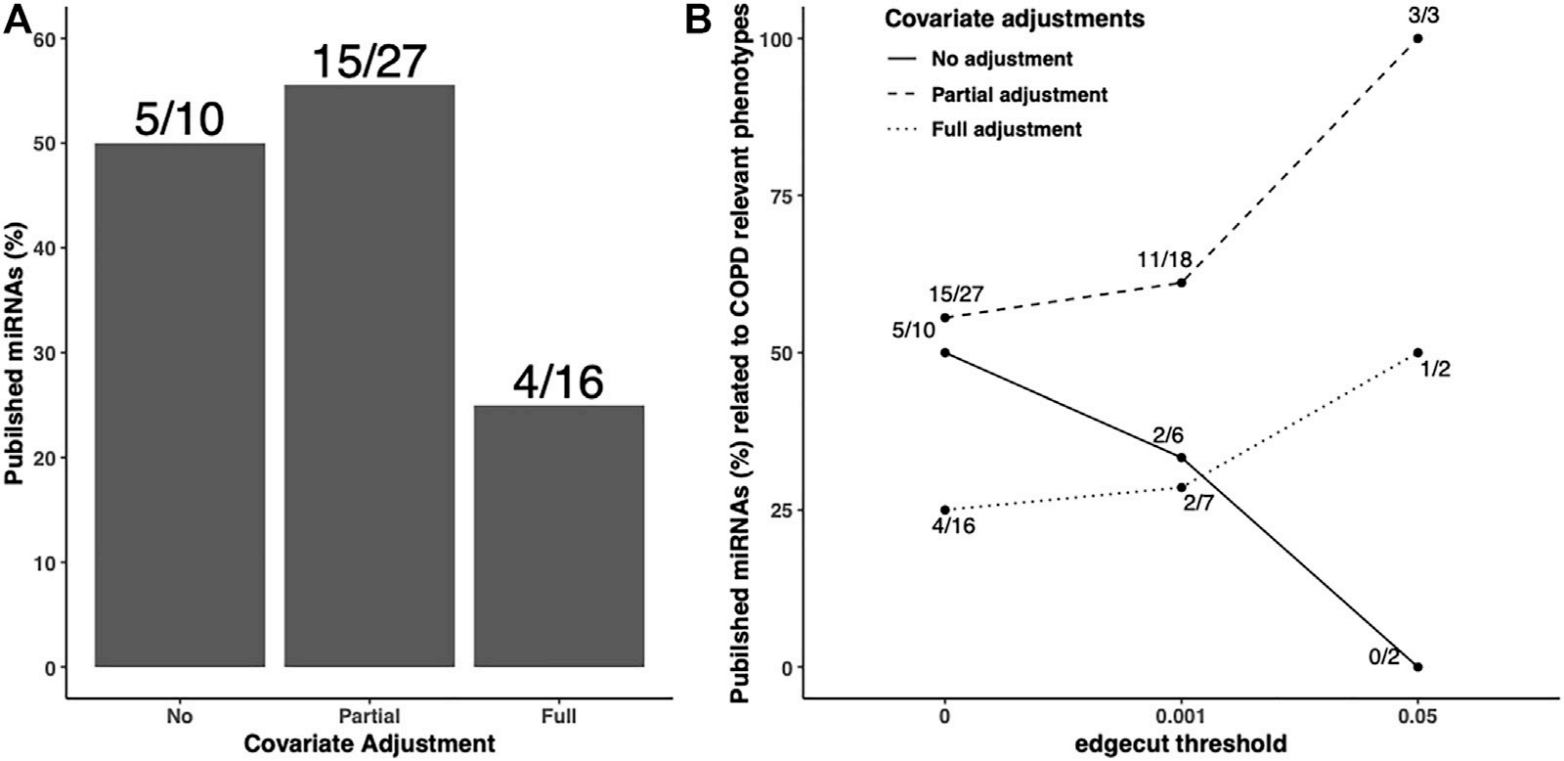
Published COPD-associated miRNA percentage in the identified FEV_1_pp-networks with different adjustment strategies. Scaled SmCCNet were applied on the unadjusted, partially adjusted, or fully adjusted miRNA and mRNA data with FEV_1_pp phenotype. To compare the quality of FEV_1_pp-associated networks, the miRNAs in the identified networks were mapped with the list of published COPD-associated miRNAs (A). The denominators on each bar are the number of miRNAs associated with FEV_1_pp with different adjustment as indicated. The numerators are the number of validated miRNAs related to COPD. We applied 0.001 and 0.05 edge thresholds to filter weak edges between miRNAs and mRNAs and the remaining miRNAs were used to calculate the published COPD-associated miRNA percentage in unadjusted, partially adjusted, and fully adjusted.

In addition to the predicted/validated miRNA–mRNA pairs, we also used another metric, published miRNAs related to COPD relevant phenotypes, to compare the adjustment strategies. We found 10, 27, and 16 miRNAs in unadjusted, partially adjusted, or fully adjusted network analysis, respectively (Figure 4A). Among the identified COPD-associated miRNAs in three covariate adjustment strategies, we found 5, 15, and 4 have been published correspondingly. The partially-adjusted networks had the highest percentage of published miRNAs percentages (55.5%) regardless of edge cutoff (Figure 4B).

Taken together, compared with unadjusted control, the partial adjustment but not full adjustment improved FEV_1_pp-associated network estimation in terms of prediction accuracy mapping to published COPD-related miRNAs. In addition, we performed a similar analysis with percent emphysema networks based on the three different covariate adjustment methods (Supplementary Figure S3-S4). Compared with unadjusted control, both partial adjustment and full adjustment had higher percentages of accuracies of miRNA–mRNA pairs when mapping with predicted and validated data bases. We also found that the partial adjustment but not full adjustment improved percent emphysema-associated network estimation in terms of prediction accuracy mapping against the published COPD-related miRNAs. Our study demonstrated that partial covariate adjustment for age, sex, and race, in addition to CT-scanner for percent of emphysema, improved network identification when compared with no covariate adjustment. However, further adjusting for smoking and blood cell composition sometimes weakened the power of identifying networks associated with COPD. Therefore, we focused the network construction on the partially adjusted data and clinical phenotypes.

### microRNA–mRNA Networks Associated With FEV_1_pp or Percent Emphysema

With partial covariate adjustment, we found three miRNA–mRNA networks associated with FEV_1_pp and three miRNA–mRNA networks associated with percent emphysema (Table 3). For each module, we trimmed the networks with increasing edge threshold. The number of nodes, the ratio of miRNA and mRNAs, and the correlations of 1st PC of trimmed networks and phenotypes were considered together to select the edge threshold for optimal network pruning. We focus on Module 3, which has the highest absolute correlation to FEV_1_pp and Module 6, which has highest absolute correlation to percent emphysema (Table 3).

**TABLE 3:** Summary of miRNA–mRNA networks associated with COPD.

Module number	Associated phenotypes	Absolute correlations	*p*-Values	No. of miRNA	No. of mRNA	Module size
1	FEV_1_pp	0.0979	0.049	6	29	35
2	FEV_1_pp	0.1543	0.0019	5	21	26
3	FEV_1_pp	0.3341	5.40E-12	2	28	30
4	% emphysema	0.3202	4.40E-11	4	67	71
5	% emphysema	0.1885	0.00014	4	126	130
6	% emphysema	0.4524	8.80E-22	5	81	86

Note: Absolute correlations: the absolute value of Pearson correlation coefficient between the 1st PC of the trimmed network and phenotypes [FEV_1_pp or percent (%) emphysema]. *p*-Values: *p* values of the Pearson correlation test. The significance level of the correlation between PC and phenotype is 0.05.

**FIGURE 5:**
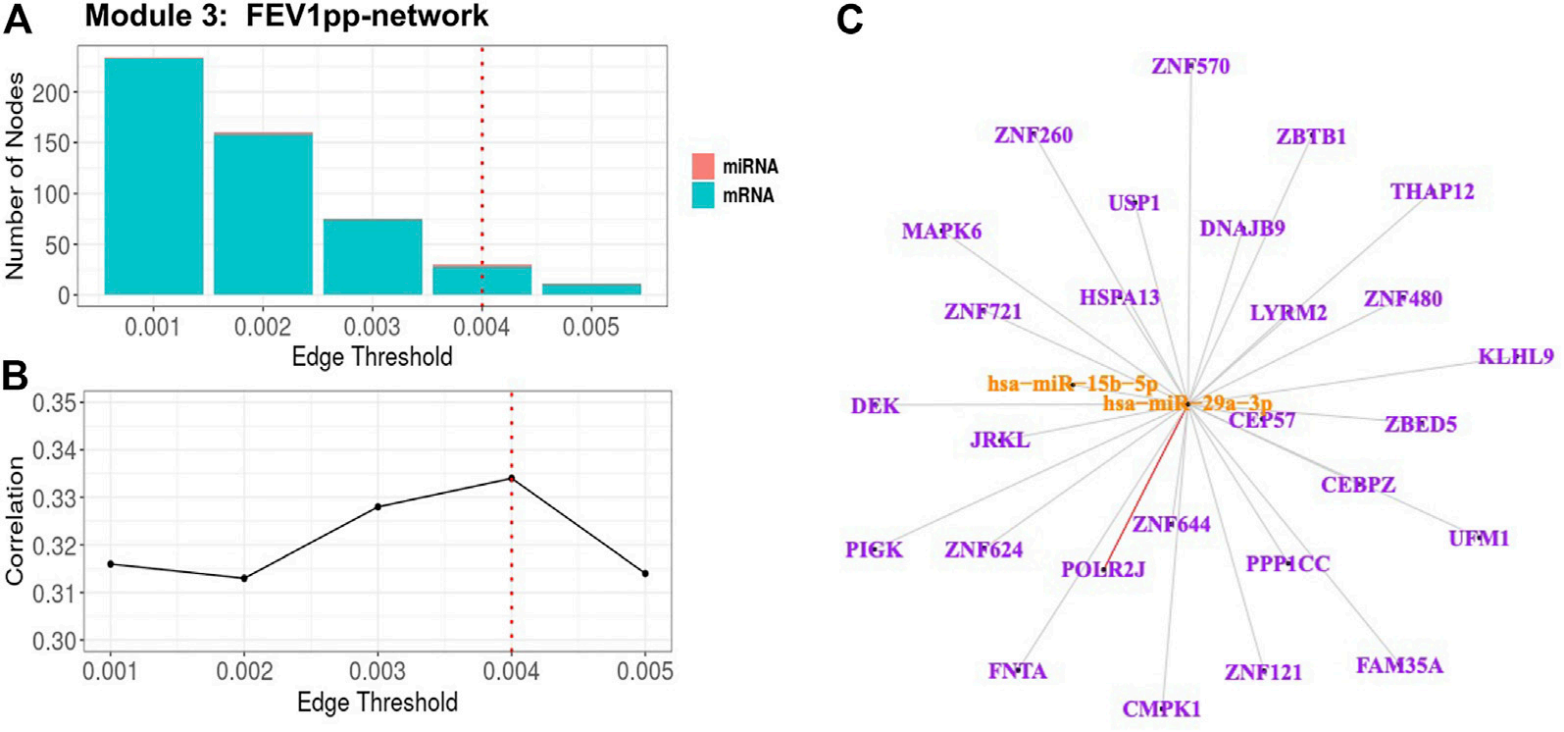
FEV_1_pp-associated miRNA–mRNA network (Module 3 in Table 3) pruning with grid search. The edge threshold candidates were defined by the distribution of the edges in the raw network. **(A)** The number of nodes including miRNA (red) and mRNA (light blue) with different edge thresholds. **(B)** The absolute correlation between FEV_1_pp and the 1st PC (i.e., first principal component) of the trimmed network under different edge thresholds. The red dotted line indicated the chosen optimal edge threshold (0.004) for network trimming, which achieved a maximum correlation (0.33) between FEV_1_pp and the 1st PC. **(C)** The trimmed miRNA–mRNA network (module 3 in Table 3) with the optimal edge threshold (0.004). The orange nodes denote miRNAs while the purple nodes denote genes. The signs of edges are based on the correlation of the original expression data between the nodes. Red and blue edges represent negative and positive correlations respectively. Edge thickness corresponds to the strength of relationship between the nodes based on the canonical weights.

For Module 3 related to FEV_1_pp (Table 3), the edge thresholds were selected with a grid search (Figure 5A). The maximum absolute correlation between the 1st PC of the trimmed network and FEV_1_pp was achieved when the edge threshold was set to 0.004 (Figure 5B). The optimal edge threshold was chosen as 0.004, which leads to a trimmed network including two miRNAs and 28 mRNAs (Table 3). The absolute correlation between the 1st PC of this trimmed network and FEV_1_pp is 0.33 (*p* = 5.40E-12). The trimmed graph is presented in Figure 5C, where hsa-miR-15b-5p and hsa-miR-29a-3p serve as two hubs in the networks.

**FIGURE 6:**
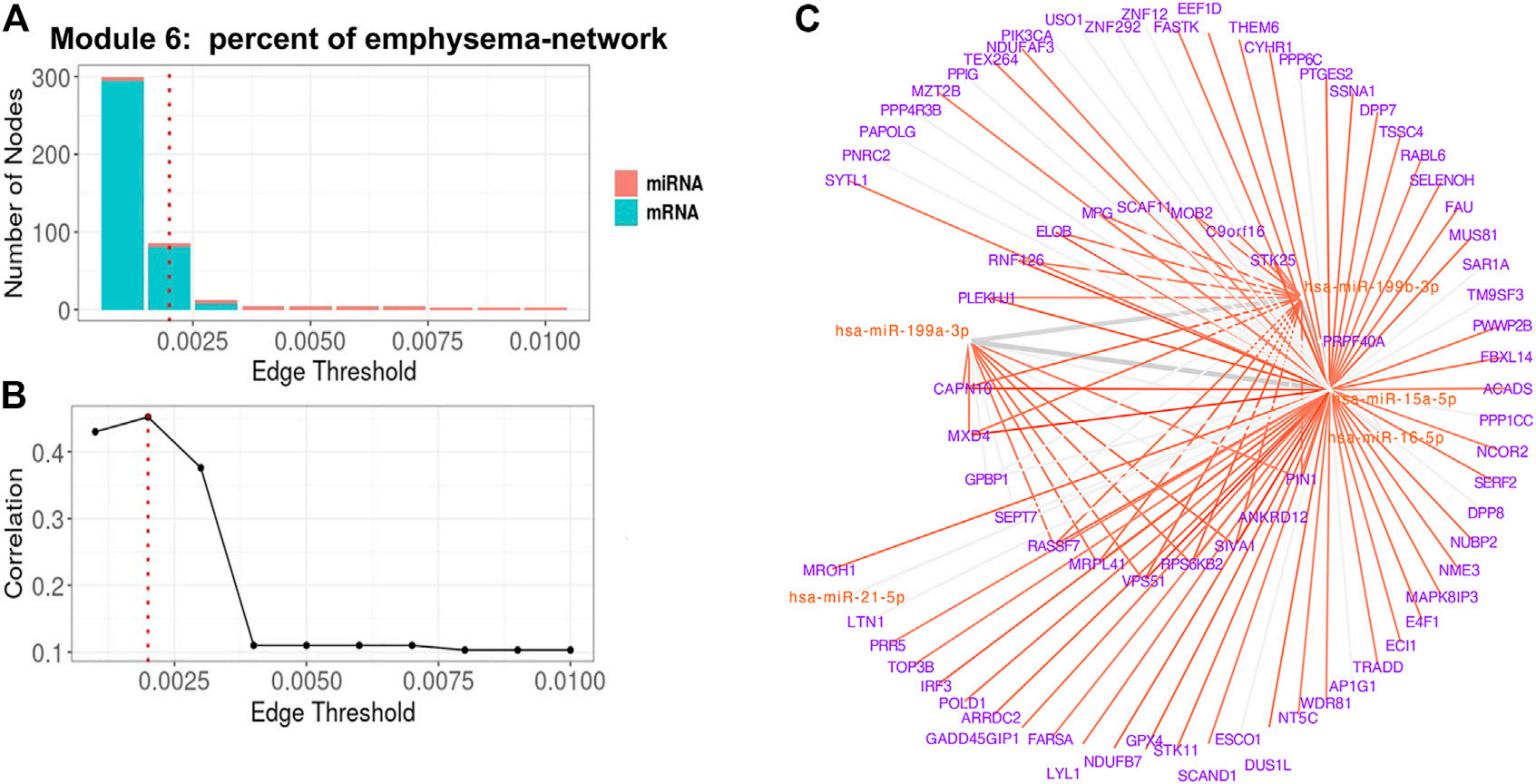
Percent of emphysema-associated miRNA–mRNA network (Module 6 in Table 3) pruning with grid search. The edge threshold candidates were defined by the distribution of the edges in the raw network. **(A)** The number of nodes including miRNA (red) and mRNA (light blue) with different edge thresholds. **(B)** The absolute correlation between percent emphysema and the 1st PC (i.e., first principal component) of the trimmed network under different edge thresholds. The red dotted line indicated the chosen optimal edge threshold (0.002) for network trimming, which achieved a maximum correlation (0.35) between FEV_1_pp and the 1st PC. **(C)** The trimmed miRNA–mRNA network (module 6 in Table 3) with the optimal edge threshold (0.002). The signs of edges are based on the correlation of the original expression data between the nodes. Red and blue edges represent negative and positive correlations respectively. Edge thickness corresponds to the relationships between the nodes based on the canonical weights.

For Module 6 related to percent emphysema (Table 3), the absolute correlation between the 1st PC of the trimmed network and percent emphysema first increased and then decreased when the edge threshold increased (Figures 6A,B). The optimal edge threshold was chosen as 0.002, which leads to a trimmed network including 5 miRNAs and 81 mRNAs (Table 3). The absolute correlation between the 1st PC of this trimmed network and percent emphysema is 0.45 (*p* = 8.80E-22). The trimmed graph is presented in Figure 6C, where hsa-miR-15a-5p, hsa-miR-16-5p, hsa-miR-199b-3p, and hsa-miR-199a-3p serve as hubs in the pruned networks.

We also pruned the other four identified modules with similar strategies. The correlations between the FEV_1_pp or percent emphysema and trimmed modules are included in the supplement (Supplementary Figure S5-S8).

### Enrichment Analysis on the Pruned COPD Networks

We performed classical gene ontology (GO) enrichment analysis on the gene nodes in the FEV_1_pp-associated and percent emphysema-associated networks to identify important pathways related to COPD. In the top FEV_1_pp-associated network (Module 3 in Table 3), we found that the enriched biological processes include regulation of transcription, DNA repair, immunity, cellular response, and metabolic regulation (Supplementary Table S3). In classical GO enrichment analysis, all genes annotated to GO terms are automatically annotated to its parents and it could lead to redundancy. To avoid redundancy, we also took into account the GO hierarchy and performed conditional enrichment tests. We that found regulation of transcription RNA polymerase II, fibroblast growth factor receptor signaling pathway, and joining and regulation of DNA repair pathways are conditionally enriched in the FEV_1_pp-associated network (Supplementary Table S4).

In the top emphysema-associated network (Module 6 in Table 3), we found that the enriched biological processes include cytosolic transport, TOR signaling, regulation of translation, and metabolic regulation (Supplementary Table S5). In the conditional GO enrichment analysis, we found that the apoptotic process, TOR signaling, COPII vesicle coating, glutathione metabolic process, endosome transportation, and type I interferon production are significantly enriched in this emphysema-associated network (Supplementary Table S6).

### Network Analysis Identified mRNAs and miRNAs Overlooked in Single -Omic Analysis

In the above three identified FEV_1_pp-related networks, there are 74 gene nodes and 13 miRNA nodes. To compare the nodes in the identified networks with the biomarkers in a single -omics study, we performed Pearson correlation testing on mRNAs/miRNAs with FEV_1_pp. With Pearson correlation analysis on the single omics data, we found 32 miRNAs significantly associated with FEV_1_pp. Of the 13 miRNAs in the FEV_1_pp networks identified through SmCCNet, only one of them (“hsa-miR-145-5p”) was significantly correlated with FEV_1_pp in the single -omics analysis. Of the 74 genes in the identified FEV_1_pp networks, only 12 of them were significantly correlated with FEV_1_pp in the Pearson correlation analysis.

In the three identified percent emphysema-related networks, there are 269 gene nodes and 13 miRNA nodes. With Pearson correlation analysis on the single omics data, we found 25 miRNAs significantly associated with percent emphysema. Of the 13 miRNAs in the percent emphysema networks identified through SmCCNet, none of them was significantly correlated with percent emphysema in the single -omics analysis. Of the 269 genes in the identified percent emphysema-related networks, 187 of them were significantly correlated with percent emphysema in the Pearson correlation analysis.

## Discussion

For studies of complex traits in human, it is important to correct for baseline characteristics, referred to as covariate adjustment. In particular, the expression of mRNA and miRNA in blood from human studies may be influenced by many covariates such as sex, race, white blood cell count, and percentages. However, there is a lack of consensus on the best way to account for covariates, what covariates need to be adjusted, and the effect of covariate adjustment on the data. Therefore, we applied SmCCNet to unadjusted, partially adjusted (age, sex and race, in addition to CT-scanner for percent emphysema), and fully-adjusted (age, sex, race, CT-scanner for percent emphysema, current smoking status and WBC percentages) expression data in parallel. Evaluation of optimal covariate adjustment in identified networks was based on the prediction accuracy of miRNAs compared to published COPD-related miRNAs and measuring the frequency of validated/predicted miRNA–mRNA pairs. We found that the partial adjustment, but not full adjustment improved both the FEV_1_pp-associated network and percent emphysema-associated network estimation based on this evaluation strategy. However, further adjusting for smoking and white blood cell percentages sometimes weakened the power of identifying networks associated with COPD, which may be due to their correlations with the COPD phenotypes. Of note, the fraction of validated miRNA–mRNA pairs in the discovered miRNA–mRNA pairs is low, but it is much larger than what would be expected by chance. Taking all miRNA–mRNA pairs in the multiMiR database for the miRNA and genes in our analysis, there are 4,399 predicted and 3060 validated miRNA–mRNA pairs. The union of these two resources are 6456 miRNA–mRNA pairs. In our dataset, there are 6,412,400 potential miRNA–mRNA pairs [9,430 (# of mRNAs in our data) * 683 (# of miRNAs in data)]. Therefore, the percentage of validated/predicted pairs possible is 6456/6,412,400 (∼0.1%). However, our approach identified a smaller set of miRNA–mRNA pairs, which had ∼8.0% overlap with the multiMiR pairs, indicating that we are finding pairs that are enriched in predicted/validated miRNA–mRNA interactions from multiMiR compared to randomly selected pairs of miRNA and mRNA from our data.

With partial covariate adjustment, we found three miRNA–mRNA networks associated with FEV_1_pp and another three miRNA–mRNA networks associated with percent emphysema. The identified miRNA–mRNA networks provide additional information on COPD-related traits that complements biomarkers identified through a single-omics analyses. The FEV_1_pp miRNA–mRNA networks and percent emphysema network share three genes (CAPZA1, CEP57, and SLC15A3) and eight miRNAs including hsa-miR-145-5p, hsa-miR-223-3p, hsa-miR-26b-3p, hsa-miR-338-5p, hsa-miR-1275, hsa-miR-150-3p, hsa-miR-150-5p, and hsa-miR-342-3p. One miRNA–mRNA network consists of two miRNAs and 28 mRNAs which had a strong correlation (*r* = 0.33) to FEV_1_ percent predicted, where hsa-miR-15b-5p and hsa-miR-29a-3p are hubs. hsa-miR-15b-5p and hsa-miR-29a-3p have been recently identified as biomarkers for fibrosis and lung diseases including COPD (Sessa and Hata, 2013; Budding et al., 2017). We also found a network of five miRNAs and 81 mRNAs that had a strong correlation (*r* = 0.45) to percent emphysema, where hsa-miR-15a-5p, hsa-miR-16-5p, and hsa-miR-199b-3p are hubs. hsa-miR-15a-5p is one of the top five miRNAs in 151 differentially expressed miRNAs that target differentially expressed mRNAs related to COPD (Qian et al., 2018). hsa-miR-15a might prevent the progression of acute exacerbations of COPD by inhibition of *Wnt* signaling (Reuter et al., 2016). The enrichment of the Cadherin/Wnt/Catenin pathways in FEV_1_pp networks was also found in our pilot study with much smaller sample size (Shi et al., 2019). For another hub hsa-miR-199a-5p, its expression in lung is diminished in COPD patients (Hassan et al., 2014). Decreased expression of hsa-miR-199a-5p leads to an intensification of the unfolded protein responses (UPRs) and contributes to lung cell apoptosis and lung inflammation. Many genes in the identified networks have also been shown to have associations with COPD development. For example, IFN regulatory factor-3 (IRF-3) plays an essential role in COPD exacerbation (Ishii et al., 2017). The GO enrichment analysis suggests that regulation of transcription, DNA repair, immune response, cellular response to unfolded protein, and metabolic regulation are enriched in the identified FEV_1_pp network. The cytosolic transport (RNF126, PLEKHJ1, VPS51, and AP1G1), target of rapamycin (mTOR) signaling (PIK3CA, STK11, RPS6KB2, and PRR5), regulation of translation, metabolic regulation, and apoptosis are involved in the percent emphysema-associated network. It was recently reported that diminished DNA repair underlies the complex and heterogeneous manifestations of COPD (Sauler et al., 2018) and that mTOR plays a major role in driving lung cell senescence and lung alterations in COPD (Houssaini et al., 2018). Recent studies also demonstrate that metabolic reprogramming occurs in COPD patients and metabolic dysregulation impacts cellular functions and contributes to the pathogenesis and progression of these diseases (Zhao et al., 2018). In addition, many animal models and human studies support an important role for apoptosis in the pathogenesis of COPD and emphysema (Demedts et al., 2006; Comer et al., 2013; Yoshida et al., 2019). Of note, many of the genes and miRNAs mentioned above in the identified COPD-related networks were not significantly associated with COPD phenotypes and were therefore overlooked in a single -omics analysis. These results demonstrate how identifying miRNA–mRNA networks through SmCCNet on multiple-omics data provides additional information for COPD-related traits that complement biomarkers identified through a single-omics analyses.

Although we identified several networks related to COPD phenotypes, there are some limitations in this study. One limitation in covariate adjustment evaluation is that the published COPD-related miRNAs were collected not only in blood but also other samples such as lung tissue. Ideally, it would be useful to have a large list of blood-specific COPD-related miRNAs for evaluation since our transcriptomic data were from peripheral blood samples, but existing miRNA studies in blood for COPD are limited, and our study is one of the largest. Another limitation is that our ground truth for miRNA–mRNA validated target pairs might not be complete or specific to COPD. The other limitation is that we only evaluated the negative interactions between miRNAs and mRNAs in the identified networks, which ignores the potential positive indirect relationships between miRNAs and mRNAs. In addition, GO enrichment analysis resulted in the identification of inflammation pathways. Although this pathway is not specific to COPD, inflammatory responses are relevant to COPD and demonstrates the reasonable network findings in this study. However, in addition to these more general enrichment results, we also identified other important pathways and miRNA, which furthers our understanding of more specific mechanisms associated with COPD, in addition to important regulatory roles of miRNA during COPD pathogenesis. Finally, we did not develop a prediction model of clinical traits and instead focused on discovery of integrated microRNA–mRNA networks underlying COPD outcomes. Risk score prediction could be an important future direction for clinical translation of our findings when larger sample sizes, in addition to replication cohorts, are available to create a reproducible and robust risk score.

## Conclusion

With partial covariate adjustment, we found six miRNA–mRNA networks associated with COPD-related phenotypes. Many genes and miRNAs in the identified networks have been shown to have associations with COPD development and progression. We found the identified networks to be enriched in many biological processes including DNA repair, apoptosis, mTOR signaling, and metabolic regulation, which have been reported to contribute to the pathogenesis of COPD and emphysema. The identified miRNA–mRNA networks provide additional information on COPD-related traits that complement biomarkers identified through a single-omics analysis, in addition to highlighting the potential role of miRNA in regulating certain COPD related gene and pathways.

## Data Availability

Clinical Data are available through COPDGene (https://www.ncbi.nlm.nih.gov/gap/, ID: phs000179.v6.p2). RNA-Seq data are available through dbGaP (https://www.ncbi.nlm.nih.gov/gap/, ID: phs000765.v3.p2). The miRNA data will be available through dbGap as well (https://www.ncbi.nlm.nih.gov/gap/).
