# Supplementary material for: Identifying miRNA-mRNA Networks Associated With COPD Phenotypes
Source: Front Genet. 2021 Oct 28;12:748356. doi: 10.3389/fgene.2021.748356 (PMC8581181; doi:10.3389/fgene.2021.748356)
Supplement: Supplementary file 2 [file DataSheet1.docx]

**Supplementary method**

**Scaled SmCCNet**

We applied a scaled version of SmCCNet (i.e., *a, b,* and *c* are not all equal) to prioritize the correlations between the miRNA data and the phenotype of interest.

($w_{1}, w_{2})=\arg{max}_{\tilde{w}_{1}, \tilde{w}_{2}}(a\tilde{w}_{1}^{T}X_{1}^{T}X_{2}\tilde{w}_{2}+b\tilde{w}_{1}^{T}X_{1}^{T}Y+c\tilde{w}_{2}^{T}X_{2}^{T}Y)$

We systematically searched the scaling factors (*a, b, c*) with different relative ratios (1:1:1 to 1:1:20) under the constraint that all three factors sum up to 3 and *a* = *b*. For each set of scaling factors, the sparse penalty parameters (*l_1_*, *l_2_*) were chosen through a 4-fold cross validation to find the penalty pair that minimized prediction error. All penalty pairs from the set were also tested in a grid search to find the optimal pair (*l_1_*, *l_2_*) (Shi et al., 2019). The penalty parameters determine how many miRNAs (*l_1_*) and mRNAs (*l_2_*) are in the final results.

Scaling constants in the grid search were evaluated using the proposed criterion to identify which value yielded the best network results.

($a,b, c)=\arg{{max}_{\tilde{a}, \tilde{b}, \tilde{c}}}(Canonical Correlation of test-error of prediction)*\left( \frac{c}{a} \right)^{\frac{1}{3}}*{(\frac{l_{1}}{l_{2}})}^{3}$

where *l_1_* and *l_2_* are sparse penalty constants for the number of mRNAs and miRNAs.

**Supplementary results**

**Table S1. Summary of selected scaling constants**

|  | **Scaling constants** | | |
| --- | --- | --- | --- |
|  | a | b | c |
| **FEV_1_pp SmCCNet** |  |  |  |
| unadjusted | 0.158 | 0.158 | 2.684 |
| partially-adjusted | 0.25 | 0.25 | 2.5 |
| fully-adjusted | 0.333 | 0.333 | 2.334 |
| **% emphysema SmCCNet** |  |  |  |
| unadjusted | 0.5 | 0.5 | 2 |
| partially-adjusted | 1 | 1 | 1 |
| fully-adjusted | 0.6 | 0.6 | 1.8 |

**Figure S1.** Heatmaps of module–FEV_1_pp correlations. We identified 4, 11 and 7 FEV_1_pp-associated modules in unadjusted, partially-adjusted and full-adjusted data. We computed the correlation of FEV_1_pp with the first PC1 of the module (also called module eigen gene, ME) respectively. The correlations (P values) are displayed in heatmaps.

**Figure S2.** Heatmaps of module–percent of emphysema correlations. We identified 4, 7 and 17 percent of emphysema-associated modules in unadjusted, partially-adjusted and full-adjusted data. We computed the correlation of percent emphysema with the first PC1 of the module (also called module eigen gene, ME). The correlations (P values) are displayed in heatmaps.

**Table S2. Summary of emphysema-related modules**


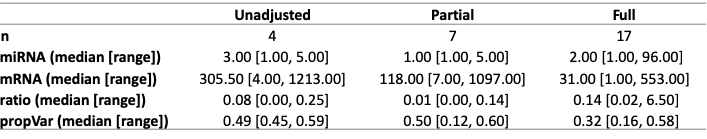


Notes: propVar, percent variance explained; range displayed with [minimum, maximum].


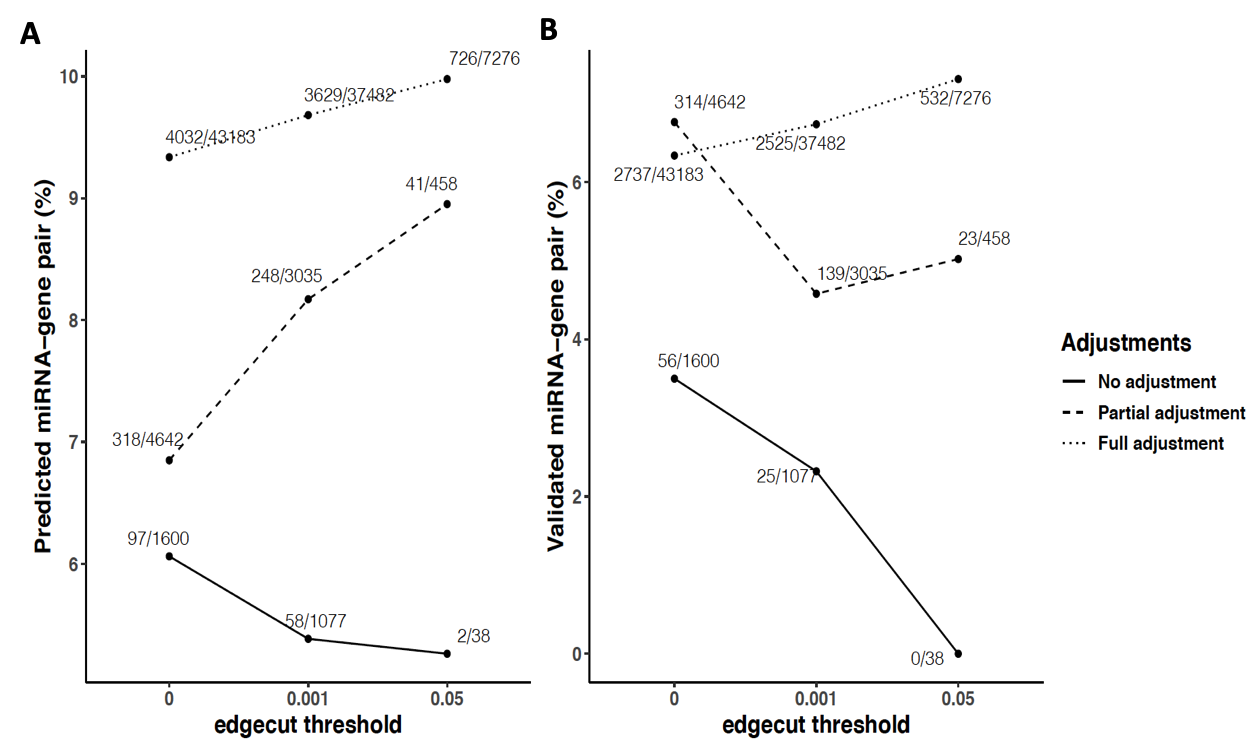


**Figure S3.** Predicted/validated miRNA-mRNA pairs percentage in the identified emphysema-networks with different adjustment strategies. Weighted SmCCNet were applied on the unadjusted, partially-adjusted or full-adjusted miRNA and mRNA data with the percent of emphysema phenotype. To compare the quality of percent of emphysema-associated networks, the pairs of miRNA and mRNA (gene) with negative correlation in the identified networks were queried in multiple microRNAs/targets databases, including predicted microRNA-target databases and validated microRNA-target databases. The queries were performed with the *multiMiR* R package as discussed in the Methods. The ratio of predicted (A) or validated (B) pairs in databases and total pairs in the constructed networks. We applied 0.001 and 0.05 edge thresholds to filter weak edges between miRNAs and mRNAs, the ratio of predicted miRNA-mRNA in unadjusted, partial adjusted and full-adjustment resulted networks were updated correspondingly.

**Figure S4.** Published COPD-associated miRNA percentage in the identified emphysema networks with different adjustment strategies. Weighted SmCCNet were applied on the unadjusted, partially-adjusted or full-adjusted miRNA and mRNA data with percent of emphysema phenotype. To compare the quality of emphysema-associated networks, the miRNAs in the identified networks were mapped with the list of published COPD-associated miRNAs (A). The denominators on each bar are the number of miRNAs associated with FEV_1_pp with different adjustment as indicated. The nominators are the number of validated miRNAs related to COPD. We applied 0.001 and 0.05 edge thresholds to filter weak edges between miRNAs and mRNAs and the remained miRNAs was used to calculate the published COPD-associated miRNA percentage in unadjusted, partial adjusted and full-adjustment.


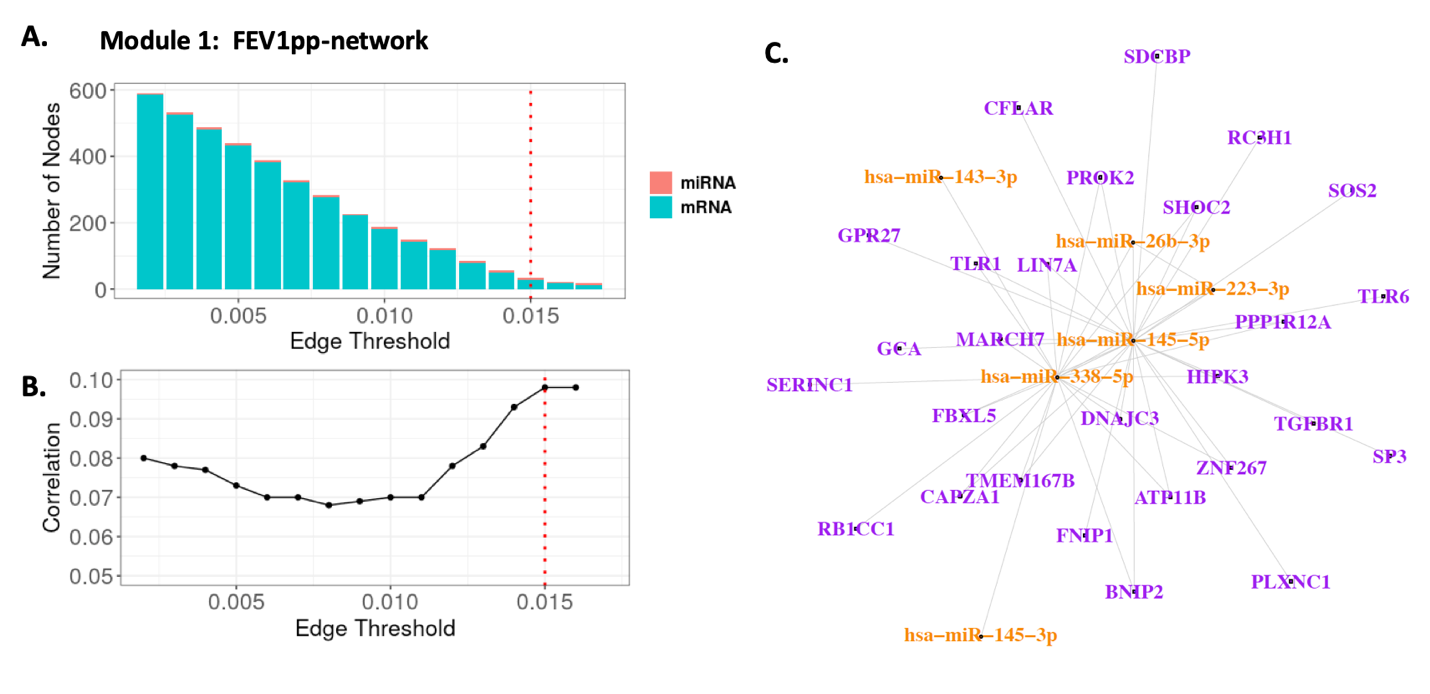


**Figure S5.** FEV_1_pp-associated miRNA-mRNA network (module 1 in Table 3) pruning with grid search. The edge threshold candidates were defined by the distribution of the edges in the raw network. (A) The number of nodes including miRNA(red) and mRNA (light blue) with different edge thresholds. (B) The absolute correlation between FEV_1_pp and the eigen gene (i.e., first principal component) of the trimmed network under different edge thresholds. The red dotted line indicated the chosen optimal edge threshold (0.015) for network trimming, which achieved a maximum correlation (0.098) between FEV_1_pp and the eigen gene. (C) The trimmed miRNA-mRNA network (module 1 in Table 3) with the optimal edge threshold (0.015) The orange nodes denote miRNAs while the purple nodes represent genes. The signs of edges are based on the correlation of the original expression data between the nodes. Red and blue edges represent negative and positive correlations respectively.


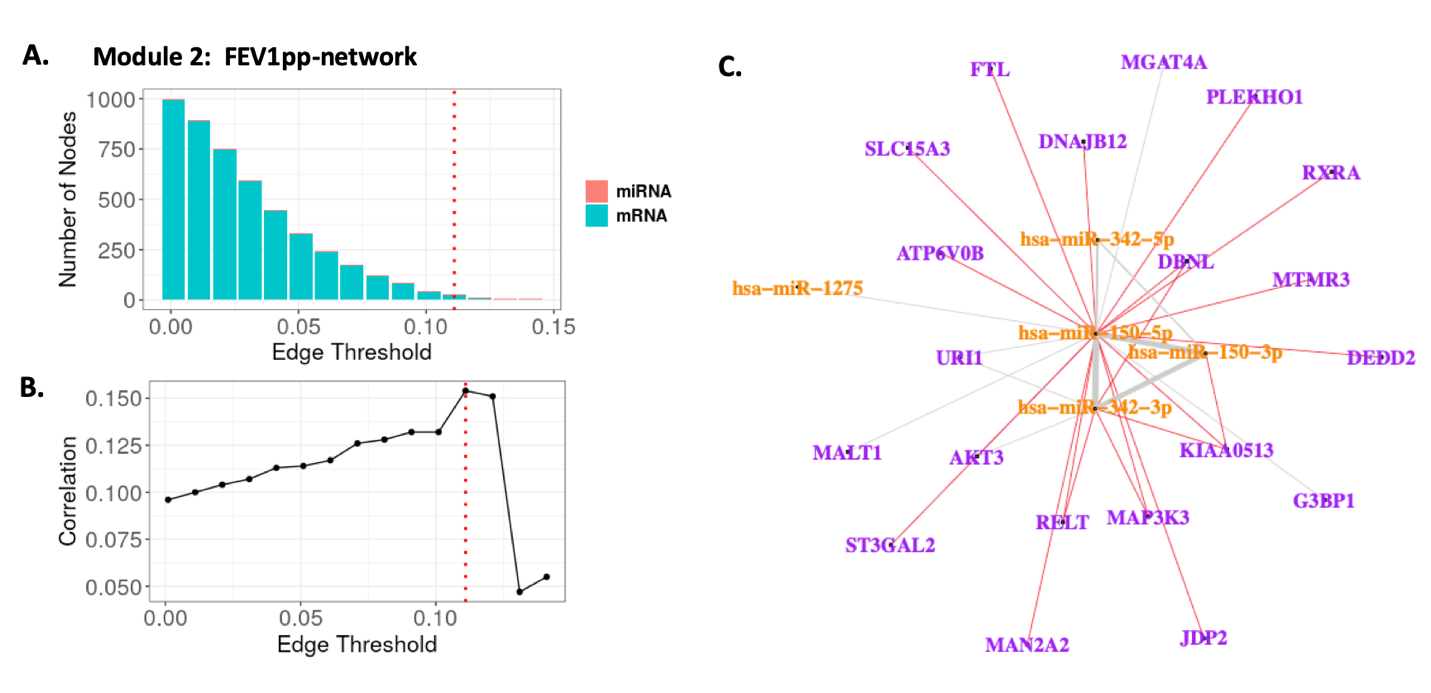


**Figure S6.** FEV_1_pp-associated miRNA-mRNA network (module 2 in Table 3) pruning with grid search. The edge threshold candidates were defined by the distribution of the edges in the raw network. (A) The number of nodes including miRNA(red) and mRNA (light blue) with different edge thresholds. (B) The absolute correlation between FEV_1_pp and the eigen gene (i.e., first principal component) of the trimmed network under different edge thresholds. The red dotted line indicated the chosen optimal edge threshold (0.111) for network trimming, which achieved a maximum correlation (0.153) between FEV_1_pp and the eigen gene. (C) The trimmed miRNA-mRNA network (module 2 in Table 3) with the optimal edge threshold (0.111). The orange nodes denote miRNAs while the purple nodes denote genes. The signs of edges are based on the correlation of the original expression data between the nodes. Red and blue edges represent negative and positive correlations respectively.


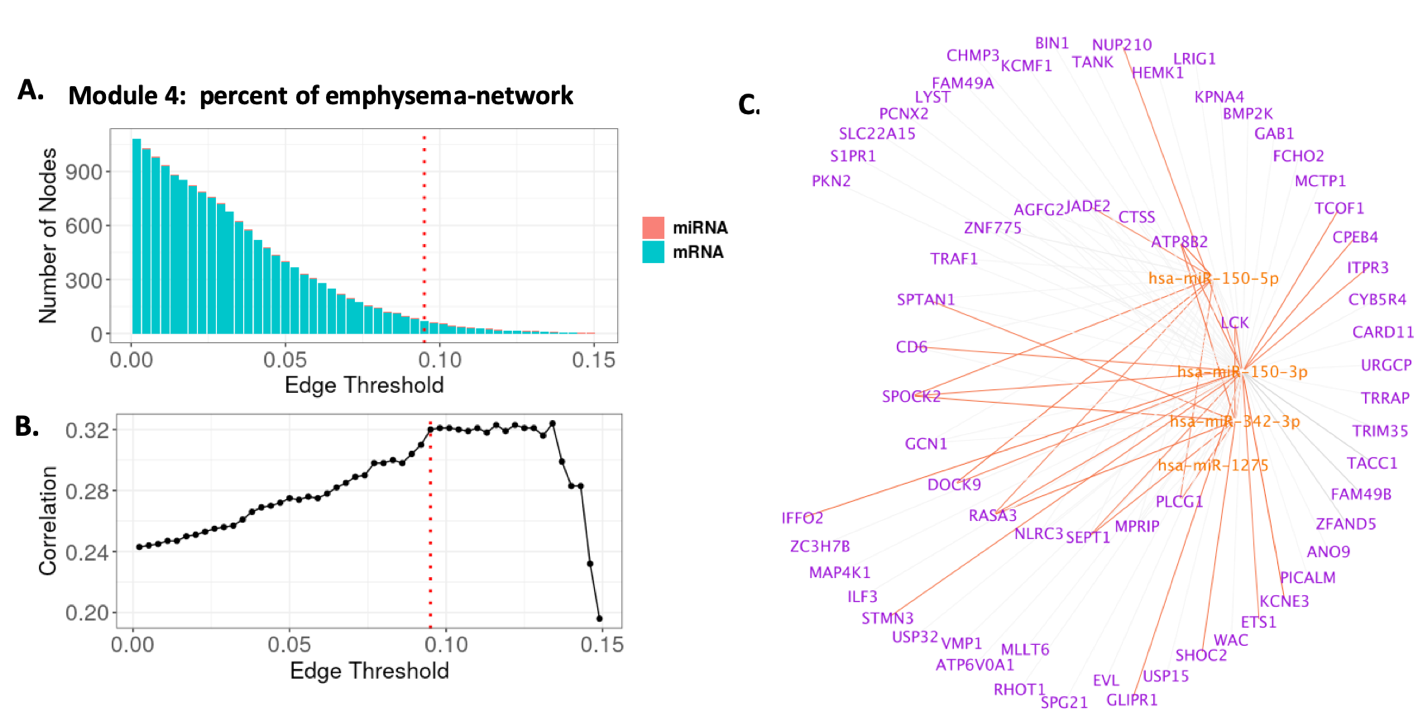

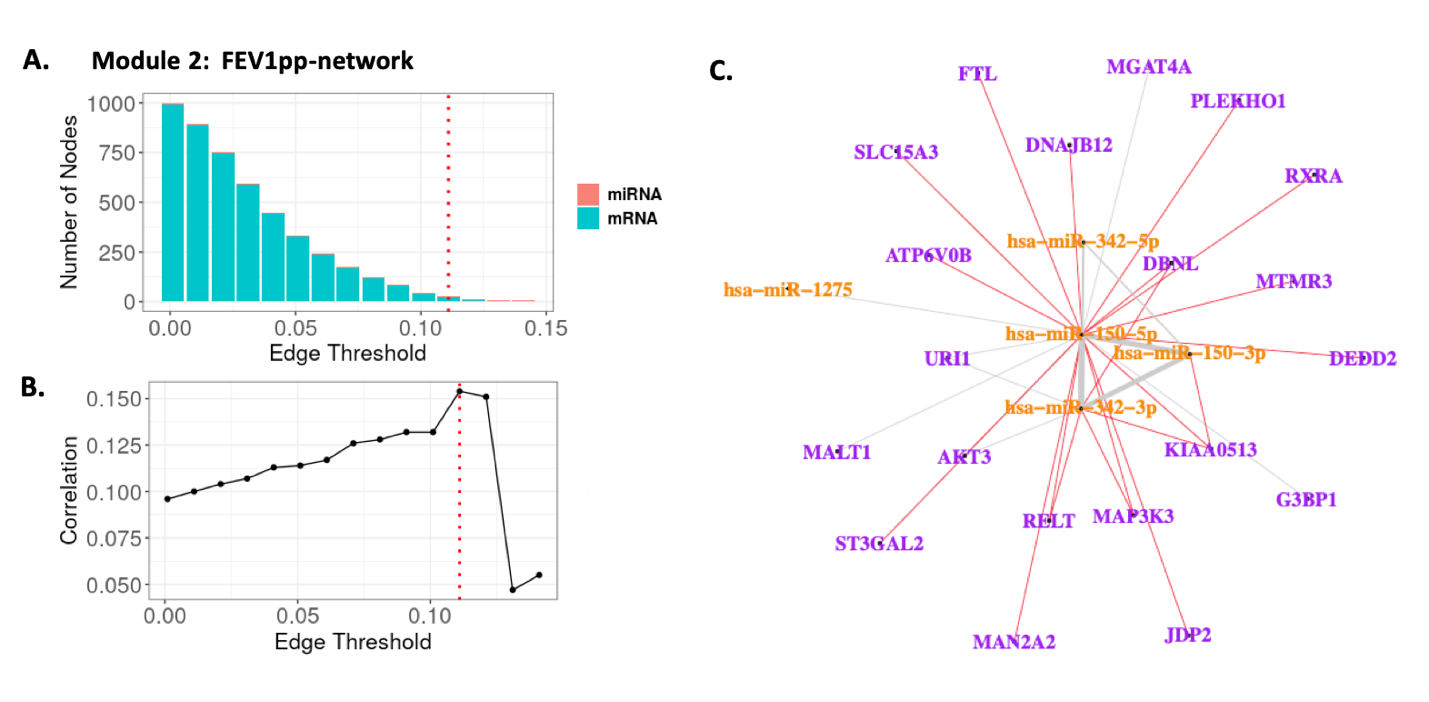


**Figure S7.** Percent of emphysema-associated miRNA-mRNA network (module 4 in Table 3) pruning with grid search. The edge threshold candidates were defined by the distribution of the edges in the raw network. (A) The number of nodes including miRNA(red) and mRNA (light blue) with different edge thresholds. (B) The absolute correlation between percent of emphysema and the eigen gene (i.e., first PC) of the trimmed network under different edge thresholds. The red dotted line indicated the chosen optimal edge threshold (0.095) for network trimming, which achieved a maximum correlation (0.32) between FEV_1_pp and the eigen gene. (C) The trimmed miRNA-mRNA network (module 4 in Table 3) with the optimal edge threshold (0.095). The orange nodes denote miRNAs while the purple nodes denote genes. The signs of edges are based on the correlation of the original expression data between the nodes. Red and blue edges represent negative and positive correlations respectively.


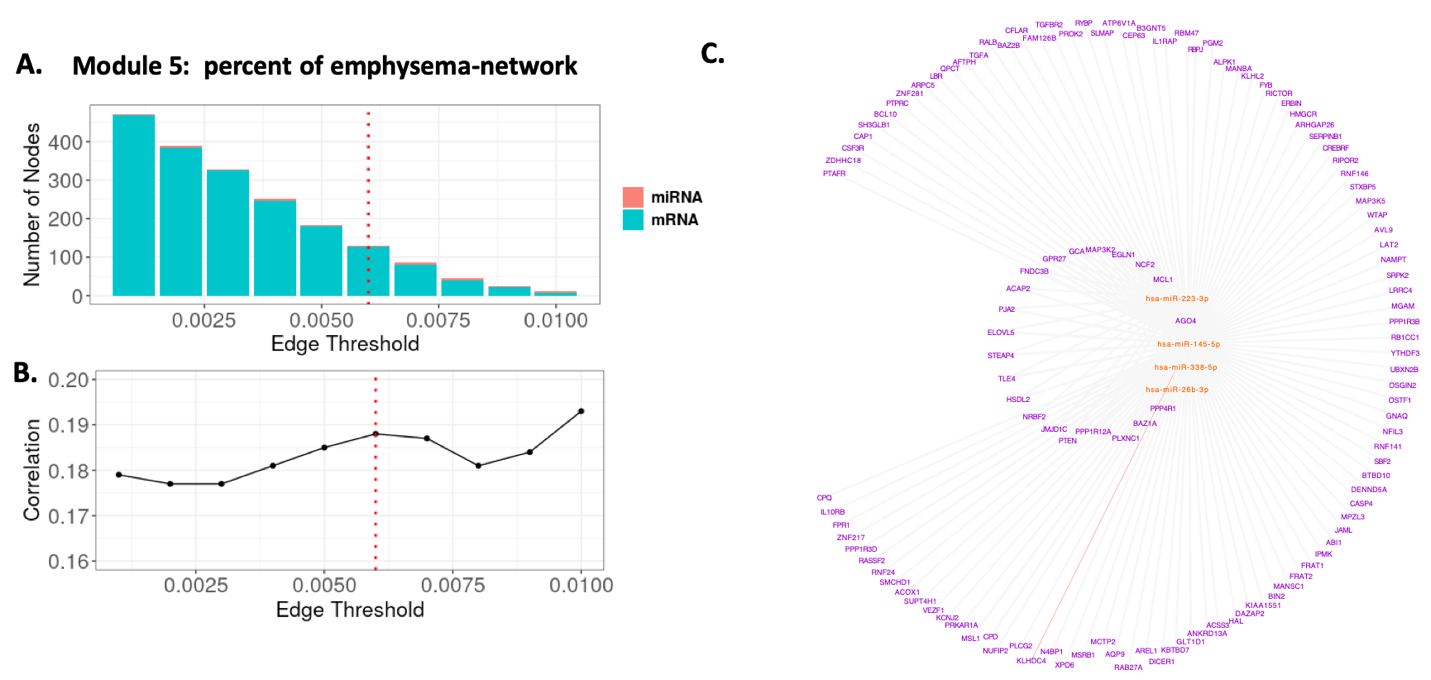


**Figure S8.** Percent of emphysema-associated miRNA-mRNA network (module 5 in Table 3) pruning with grid search. The edge threshold candidates were defined by the distribution of the edges in the raw network. (A) The number of nodes including miRNA(red) and mRNA (light blue) with different edge thresholds. (B) The absolute correlation between percent of emphysema and the eigen gene (i.e., first PC) of the trimmed network under different edge thresholds. The red dotted line indicated the chosen optimal edge threshold (0.006) for network trimming and the corresponding correlation is 0.187. (C) The trimmed miRNA-mRNA network (module 4 in Table 3) with the optimal edge threshold (0.006). The orange nodes denote miRNAs while the purple nodes denote genes. The signs of edges are based on the correlation of the original expression data between the nodes. Red and blue edges represent negative and positive correlations respectively.

**Table S3. Classical GO enrichment analysis result of**

**trimmed module 3: FEV_1_pp-network**

| **GO.ID** | **Term** | **Annotated** | **Significant** | **Expected** | **P value** |
| --- | --- | --- | --- | --- | --- |
| GO:0006366 | transcription by RNA polymerase II | 1607 | 13 | 4.93 | 0.00037 |
| GO:0006357 | regulation of transcription by RNA polymerase II | 1493 | 12 | 4.58 | 0.00077 |
| GO:0009416 | response to light stimulus | 183 | 4 | 0.56 | 0.00219 |
| GO:0006282 | regulation of DNA repair | 91 | 3 | 0.28 | 0.00262 |
| GO:0044260 | cellular macromolecule metabolic process | 4990 | 22 | 15.31 | 0.00628 |
| GO:0006351 | transcription, DNA-templated | 2167 | 13 | 6.65 | 0.00672 |
| GO:0019219 | regulation of nucleobase-containing compound metabolic process | 2450 | 14 | 7.52 | 0.00702 |
| GO:0006281 | DNA repair | 410 | 5 | 1.26 | 0.00740 |
| GO:0097659 | nucleic acid-templated transcription | 2202 | 13 | 6.76 | 0.00776 |
| GO:0032774 | RNA biosynthetic process | 2210 | 13 | 6.78 | 0.00801 |
| GO:0009314 | response to radiation | 267 | 4 | 0.82 | 0.00839 |
| GO:0034654 | nucleobase-containing compound biosynthetic process | 2522 | 14 | 7.74 | 0.00924 |
| GO:0008543 | fibroblast growth factor receptor signaling pathway | 48 | 2 | 0.15 | 0.00938 |
| GO:0018130 | heterocycle biosynthetic process | 2561 | 14 | 7.86 | 0.01068 |
| GO:0019438 | aromatic compound biosynthetic process | 2563 | 14 | 7.86 | 0.01076 |
| GO:0006355 | regulation of transcription, DNA-templated | 2032 | 12 | 6.24 | 0.01145 |
| GO:2000779 | regulation of double-strand break repair | 57 | 2 | 0.17 | 0.01305 |
| GO:2001020 | regulation of response to DNA damage stimulus | 163 | 3 | 0.5 | 0.01317 |
| GO:0060255 | regulation of macromolecule metabolic process | 3839 | 18 | 11.78 | 0.01329 |
| GO:1903506 | regulation of nucleic acid-templated transcription | 2072 | 12 | 6.36 | 0.01340 |
| GO:2001141 | regulation of RNA biosynthetic process | 2074 | 12 | 6.36 | 0.01351 |
| GO:1901362 | organic cyclic compound biosynthetic process | 2627 | 14 | 8.06 | 0.01352 |
| GO:0044344 | cellular response to fibroblast growth factor stimulus | 62 | 2 | 0.19 | 0.01531 |
| GO:0071774 | response to fibroblast growth factor | 64 | 2 | 0.2 | 0.01626 |
| GO:0010468 | regulation of gene expression | 2980 | 15 | 9.14 | 0.01663 |
| GO:0002708 | positive regulation of lymphocyte mediated immunity | 69 | 2 | 0.21 | 0.01875 |
| GO:0002824 | positive regulation of adaptive immune response based on somatic recombination of immune receptors built from immunoglobulin superfamily domains | 69 | 2 | 0.21 | 0.01875 |
| GO:0042113 | B cell activation | 191 | 3 | 0.59 | 0.02008 |
| GO:0050794 | regulation of cellular process | 5771 | 23 | 17.71 | 0.02097 |
| GO:0002821 | positive regulation of adaptive immune response | 74 | 2 | 0.23 | 0.02138 |
| GO:0006303 | double-strand break repair via nonhomologous end joining | 75 | 2 | 0.23 | 0.02193 |
| GO:0000726 | non-recombinational repair | 79 | 2 | 0.24 | 0.02416 |
| GO:0071482 | cellular response to light stimulus | 80 | 2 | 0.25 | 0.02474 |
| GO:0002377 | immunoglobulin production | 84 | 2 | 0.26 | 0.02709 |
| GO:0006807 | nitrogen compound metabolic process | 5874 | 23 | 18.02 | 0.02797 |
| GO:0051252 | regulation of RNA metabolic process | 2283 | 12 | 7.01 | 0.02846 |
| GO:0046822 | regulation of nucleocytoplasmic transport | 88 | 2 | 0.27 | 0.02952 |
| GO:0002705 | positive regulation of leukocyte mediated immunity | 93 | 2 | 0.29 | 0.03268 |
| GO:0019222 | regulation of metabolic process | 4155 | 18 | 12.75 | 0.03297 |
| GO:0030183 | B cell differentiation | 95 | 2 | 0.29 | 0.03399 |
| GO:0051260 | protein homooligomerization | 97 | 2 | 0.3 | 0.03531 |
| GO:0051606 | detection of stimulus | 97 | 2 | 0.3 | 0.03531 |
| GO:0051052 | regulation of DNA metabolic process | 247 | 3 | 0.76 | 0.03892 |
| GO:2000112 | regulation of cellular macromolecule biosynthetic process | 2383 | 12 | 7.31 | 0.03906 |
| GO:0090304 | nucleic acid metabolic process | 3279 | 15 | 10.06 | 0.04038 |
| GO:0002706 | regulation of lymphocyte mediated immunity | 105 | 2 | 0.32 | 0.04079 |
| GO:0006974 | cellular response to DNA damage stimulus | 638 | 5 | 1.96 | 0.04205 |
| GO:0002822 | regulation of adaptive immune response based on somatic recombination of immune receptors built from immunoglobulin superfamily domains | 107 | 2 | 0.33 | 0.04221 |
| GO:0043170 | macromolecule metabolic process | 5654 | 22 | 17.35 | 0.04281 |
| GO:0006139 | nucleobase-containing compound metabolic process | 3613 | 16 | 11.09 | 0.04300 |
| GO:0006259 | DNA metabolic process | 642 | 5 | 1.97 | 0.04302 |
| GO:0010556 | regulation of macromolecule biosynthetic process | 2440 | 12 | 7.49 | 0.04630 |
| GO:0050789 | regulation of biological process | 6065 | 23 | 18.61 | 0.04642 |
| GO:0009411 | response to UV | 113 | 2 | 0.35 | 0.04658 |
| GO:0034620 | cellular response to unfolded protein | 115 | 2 | 0.35 | 0.04807 |

**Table S4. Conditional GO enrichment analysis result of**

**trimmed module 3: FEV_1_pp-network**

| **GO.ID** | **Term** | **Annotated** | **Significant** | **Expected** | **P value** |
| --- | --- | --- | --- | --- | --- |
| GO:0006357 | regulation of transcription by RNA polymerase II | 1493 | 12 | 4.58 | 2.1e-05 |
| GO:0008543 | fibroblast growth factor receptor signaling pathway | 48 | 2 | 0.15 | 0.0094 |
| GO:0006303 | double-strand break repair via nonhomologous end joining | 75 | 2 | 0.23 | 0.0219 |
| GO:0006282 | regulation of DNA repair | 91 | 3 | 0.28 | 0.0283 |
| GO:0002377 | immunoglobulin production | 84 | 2 | 0.26 | 0.0323 |
| GO:0030183 | B cell differentiation | 95 | 2 | 0.29 | 0.0340 |
| GO:0071482 | cellular response to light stimulus | 80 | 2 | 0.25 | 0.0352 |
| GO:0051260 | protein homooligomerization | 97 | 2 | 0.3 | 0.0353 |
| GO:0034620 | cellular response to unfolded protein | 115 | 2 | 0.35 | 0.0439 |
| GO:0007049 | cell cycle | 1211 | 4 | 3.72 | 0.0514 |
| GO:2000779 | regulation of double-strand break repair | 57 | 2 | 0.17 | 0.0717 |
| GO:0009581 | detection of external stimulus | 30 | 1 | 0.09 | 0.0882 |
| GO:0048538 | thymus development | 31 | 1 | 0.1 | 0.0910 |
| GO:0006458 | 'de novo' protein folding | 31 | 1 | 0.1 | 0.0910 |
| GO:0009582 | detection of abiotic stimulus | 31 | 1 | 0.1 | 0.0910 |
| GO:0009124 | nucleoside monophosphate biosynthetic process | 31 | 1 | 0.1 | 0.0910 |
| GO:0010569 | regulation of double-strand break repair via homologous recombination | 32 | 1 | 0.1 | 0.0938 |
| GO:0002711 | positive regulation of T cell mediated immunity | 32 | 1 | 0.1 | 0.0938 |
| GO:0006221 | pyrimidine nucleotide biosynthetic process | 33 | 1 | 0.1 | 0.0966 |
| GO:0090311 | regulation of protein deacetylation | 33 | 1 | 0.1 | 0.0966 |
| GO:0050434 | positive regulation of viral transcription | 34 | 1 | 0.1 | 0.0994 |
| GO:0042769 | DNA damage response, detection of DNA damage | 34 | 1 | 0.1 | 0.0994 |
| GO:0045911 | positive regulation of DNA recombination | 34 | 1 | 0.1 | 0.0994 |

**Table S5. Classical GO enrichment analysis result of**

**trimmed module 6: percent emphysema-network**

| **GO.ID** | **Term** | **Annotated** | **Significant** | **Expected** | **P value** |
| --- | --- | --- | --- | --- | --- |
| GO:0006414 | translational elongation | 118 | 5 | 0.95 | 0.0026 |
| GO:0016482 | cytosolic transport | 138 | 5 | 1.11 | 0.0050 |
| GO:0031929 | TOR signaling | 95 | 4 | 0.77 | 0.0071 |
| GO:0048207 | vesicle targeting, rough ER to cis-Golgi | 56 | 3 | 0.45 | 0.0103 |
| GO:0048208 | COPII vesicle coating | 56 | 3 | 0.45 | 0.0103 |
| GO:0006901 | vesicle coating | 60 | 3 | 0.48 | 0.0124 |
| GO:0090114 | COPII-coated vesicle budding | 63 | 3 | 0.51 | 0.0141 |
| GO:0016311 | dephosphorylation | 321 | 7 | 2.59 | 0.0145 |
| GO:0048199 | vesicle targeting, to, from or within Golgi | 64 | 3 | 0.52 | 0.0148 |
| GO:0048194 | Golgi vesicle budding | 71 | 3 | 0.57 | 0.0195 |
| GO:0042147 | retrograde transport, endosome to Golgi | 77 | 3 | 0.62 | 0.0241 |
| GO:0010720 | positive regulation of cell development | 285 | 6 | 2.3 | 0.0270 |
| GO:0006903 | vesicle targeting | 83 | 3 | 0.67 | 0.0292 |
| GO:0031346 | positive regulation of cell projection organization | 216 | 5 | 1.74 | 0.0299 |
| GO:0006220 | pyrimidine nucleotide metabolic process | 34 | 2 | 0.27 | 0.0305 |
| GO:0006518 | peptide metabolic process | 641 | 10 | 5.17 | 0.0321 |
| GO:0006470 | protein dephosphorylation | 222 | 5 | 1.79 | 0.0331 |
| GO:0006284 | base-excision repair | 36 | 2 | 0.29 | 0.0339 |
| GO:0018208 | peptidyl-proline modification | 37 | 2 | 0.3 | 0.0356 |
| GO:0032480 | negative regulation of type I interferon production | 38 | 2 | 0.31 | 0.0374 |
| GO:0006900 | vesicle budding from membrane | 93 | 3 | 0.75 | 0.0390 |
| GO:0098840 | protein transport along microtubule | 39 | 2 | 0.31 | 0.0393 |
| GO:0099118 | microtubule-based protein transport | 39 | 2 | 0.31 | 0.0393 |
| GO:0051650 | establishment of vesicle localization | 165 | 4 | 1.33 | 0.0439 |
| GO:0050769 | positive regulation of neurogenesis | 241 | 5 | 1.94 | 0.0447 |
| GO:0051648 | vesicle localization | 169 | 4 | 1.36 | 0.0473 |

**Table S6. Conditional GO enrichment analysis result of**

| **GO.ID** | **Term** | **Annotated** | | **Significant** | **Expected** | **P value** |
| --- | --- | --- | --- | --- | --- | --- |
| GO:0006915 | apoptotic process | | 1164 | 14 | 9.25 | 0.0043 |
| GO:0006414 | translational elongation | | 120 | 5 | 0.95 | 0.0051 |
| GO:0031929 | TOR signaling | | 94 | 4 | 0.75 | 0.0051 |
| GO:0048208 | COPII vesicle coating | | 56 | 3 | 0.45 | 0.0099 |
| GO:0006470 | protein dephosphorylation | | 221 | 5 | 1.76 | 0.0149 |
| GO:0006749 | glutathione metabolic process | | 30 | 2 | 0.24 | 0.0235 |
| GO:0042147 | retrograde transport, endosome to Golgi | | 78 | 3 | 0.62 | 0.0240 |
| GO:0006287 | base-excision repair, gap-filling | | 33 | 2 | 0.26 | 0.0281 |
| GO:0018208 | peptidyl-proline modification | | 36 | 2 | 0.29 | 0.0330 |
| GO:0032388 | positive regulation of intracellular transport | | 180 | 3 | 1.43 | 0.0348 |
| GO:0032480 | negative regulation of type I interferon production | | 38 | 2 | 0.3 | 0.0364 |
| GO:0010941 | regulation of cell death | | 1011 | 8 | 8.04 | 0.0398 |
| GO:0051272 | positive regulation of cellular component movement | | 300 | 2 | 2.39 | 0.0477 |
| GO:0032147 | activation of protein kinase activity | | 197 | 4 | 1.57 | 0.0515 |
| GO:0014068 | positive regulation of phosphatidylinositol 3-kinase signaling | | 49 | 2 | 0.39 | 0.0577 |
| GO:0007030 | Golgi organization | | 111 | 3 | 0.88 | 0.0581 |
| GO:0050772 | positive regulation of axonogenesis | | 50 | 2 | 0.4 | 0.0598 |
| GO:0006690 | icosanoid metabolic process | | 50 | 2 | 0.4 | 0.0598 |
| GO:0098840 | protein transport along microtubule | | 39 | 2 | 0.31 | 0.0612 |
| GO:0051668 | localization within membrane | | 51 | 2 | 0.41 | 0.0619 |
| GO:0030030 | cell projection organization | | 812 | 7 | 6.46 | 0.0623 |
| GO:0001676 | long-chain fatty acid metabolic process | | 52 | 2 | 0.41 | 0.0641 |
| GO:0006635 | fatty acid beta-oxidation | | 53 | 2 | 0.42 | 0.0662 |
| GO:0032465 | regulation of cytokinesis | | 55 | 2 | 0.44 | 0.0707 |
| GO:0030182 | neuron differentiation | | 635 | 6 | 5.05 | 0.0718 |
| GO:0045995 | regulation of embryonic development | | 56 | 2 | 0.45 | 0.0729 |
| GO:0032981 | mitochondrial respiratory chain complex I assembly | | 58 | 2 | 0.46 | 0.0775 |
| GO:0016032 | viral process | | 833 | 8 | 6.62 | 0.0825 |
| GO:0032355 | response to estradiol | | 62 | 2 | 0.49 | 0.0869 |
| GO:0043069 | negative regulation of programmed cell death | | 517 | 4 | 4.11 | 0.0927 |
| GO:0120034 | positive regulation of plasma membrane bounded cell projection assembly | | 65 | 2 | 0.52 | 0.0942 |
| GO:0043491 | protein kinase B signaling | | 127 | 3 | 1.01 | 0.0971 |
| GO:0006644 | phospholipid metabolic process | | 282 | 3 | 2.24 | 0.0988 |

**trimmed module 6: percent emphysema-network**

**Supplemental Funding and Acknowledgment**

**COPDGene Phase 3**

**Grant Support and Disclaimer**

The project described was supported by Award Number U01 HL089897 and Award Number U01 HL089856 from the National Heart, Lung, and Blood Institute. The content is solely the responsibility of the authors and does not necessarily represent the official views of the National Heart, Lung, and Blood Institute or the National Institutes of Health.

**COPD Foundation Funding**

COPDGene is also supported by the COPD Foundation through contributions made to an Industry Advisory Board that has included AstraZeneca, Bayer Pharmaceuticals, Boehringer-Ingelheim, Genentech, GlaxoSmithKline, Novartis, Pfizer, and Sunovion.

**COPDGene® Investigators – Core Units**

*Administrative Center*: James D. Crapo, MD (PI); Edwin K. Silverman, MD, PhD (PI); Barry J. Make, MD; Elizabeth A. Regan, MD, PhD

*Genetic Analysis Center*: Terri H. Beaty, PhD; Peter J. Castaldi, MD, MSc; Michael H. Cho, MD, MPH; Dawn L. DeMeo, MD, MPH; Adel El Boueiz, MD, MMSc; Marilyn G. Foreman, MD, MS; Auyon Ghosh, MD; Lystra P. Hayden, MD, MMSc; Craig P. Hersh, MD, MPH; Jacqueline Hetmanski, MS; Brian D. Hobbs, MD, MMSc; John E. Hokanson, MPH, PhD; Wonji Kim, PhD; Nan Laird, PhD; Christoph Lange, PhD; Sharon M. Lutz, PhD; Merry-Lynn McDonald, PhD; Dmitry Prokopenko, PhD; Matthew Moll, MD, MPH; Jarrett Morrow, PhD; Dandi Qiao, PhD; Elizabeth A. Regan, MD, PhD; Aabida Saferali, PhD; Phuwanat Sakornsakolpat, MD; Edwin K. Silverman, MD, PhD; Emily S. Wan, MD; Jeong Yun, MD, MPH

*Imaging Center*: Juan Pablo Centeno; Jean-Paul Charbonnier, PhD; Harvey O. Coxson, PhD; Craig J. Galban, PhD; MeiLan K. Han, MD, MS; Eric A. Hoffman, Stephen Humphries, PhD; Francine L. Jacobson, MD, MPH; Philip F. Judy, PhD; Ella A. Kazerooni, MD; Alex Kluiber; David A. Lynch, MB; Pietro Nardelli, PhD; John D. Newell, Jr., MD; Aleena Notary; Andrea Oh, MD; Elizabeth A. Regan, MD, PhD; James C. Ross, PhD; Raul San Jose Estepar, PhD; Joyce Schroeder, MD; Jered Sieren; Berend C. Stoel, PhD; Juerg Tschirren, PhD; Edwin Van Beek, MD, PhD; Bram van Ginneken, PhD; Eva van Rikxoort, PhD; Gonzalo Vegas Sanchez-Ferrero, PhD; Lucas Veitel; George R. Washko, MD; Carla G. Wilson, MS

*PFT QA Center, Salt Lake City, UT*: Robert Jensen, PhD

*Data Coordinating Center and Biostatistics, National Jewish Health, Denver, CO*: Douglas Everett, PhD; Jim Crooks, PhD; Katherine Pratte, PhD; Matt Strand, PhD; Carla G. Wilson, MS

*Epidemiology Core, University of Colorado Anschutz Medical Campus, Aurora, CO*: John E. Hokanson, MPH, PhD; Erin Austin, PhD; Gregory Kinney, MPH, PhD; Sharon M. Lutz, PhD; Kendra A. Young, PhD

*Mortality Adjudication Core*: Surya P. Bhatt, MD; Jessica Bon, MD; Alejandro A. Diaz, MD, MPH; MeiLan K. Han, MD, MS; Barry Make, MD; Susan Murray, ScD; Elizabeth Regan, MD; Xavier Soler, MD; Carla G. Wilson, MS

*Biomarker Core*: Russell P. Bowler, MD, PhD; Katerina Kechris, PhD; Farnoush Banaei-Kashani, PhD

**COPDGene® Investigators – Clinical Centers**

*Ann Arbor VA*: Jeffrey L. Curtis, MD; Perry G. Pernicano, MD

*Baylor College of Medicine, Houston, TX*: Nicola Hanania, MD, MS; Mustafa Atik, MD; Aladin Boriek, PhD; Kalpatha Guntupalli, MD; Elizabeth Guy, MD; Amit Parulekar, MD

*Brigham and Women’s Hospital, Boston, MA*: Dawn L. DeMeo, MD, MPH; Craig Hersh, MD, MPH; Francine L. Jacobson, MD, MPH; George Washko, MD

*Columbia University, New York, NY*: R. Graham Barr, MD, DrPH; John Austin, MD; Belinda D’Souza, MD; Byron Thomashow, MD

*Duke University Medical Center, Durham, NC*: Neil MacIntyre, Jr., MD; H. Page McAdams, MD; Lacey Washington, MD

*HealthPartners Research Institute, Minneapolis, MN*: Charlene McEvoy, MD, MPH; Joseph Tashjian, MD

*Johns Hopkins University, Baltimore, MD*: Robert Wise, MD; Robert Brown, MD; Nadia N. Hansel, MD, MPH; Karen Horton, MD; Allison Lambert, MD, MHS; Nirupama Putcha, MD, MHS

*Lundquist Institute for Biomedical Innovation at Harbor UCLA Medical Center, Torrance, CA*: Richard Casaburi, PhD, MD; Alessandra Adami, PhD; Matthew Budoff, MD; Hans Fischer, MD; Janos Porszasz, MD, PhD; Harry Rossiter, PhD; William Stringer, MD

*Michael E. DeBakey VAMC, Houston, TX*: Amir Sharafkhaneh, MD, PhD; Charlie Lan, DO

*Minneapolis VA*: Christine Wendt, MD; Brian Bell, MD; Ken M. Kunisaki, MD, MS

*Morehouse School of Medicine, Atlanta, GA*: Eric L. Flenaugh, MD; Hirut Gebrekristos, PhD; Mario Ponce, MD; Silanath Terpenning, MD; Gloria Westney, MD, MS

*National Jewish Health, Denver, CO*: Russell Bowler, MD, PhD; David A. Lynch, MB

*Reliant Medical Group, Worcester, MA*: Richard Rosiello, MD; David Pace, MD

*Temple University, Philadelphia, PA*: Gerard Criner, MD; David Ciccolella, MD; Francis Cordova, MD; Chandra Dass, MD; Gilbert D’Alonzo, DO; Parag Desai, MD; Michael Jacobs, PharmD; Steven Kelsen, MD, PhD; Victor Kim, MD; A. James Mamary, MD; Nathaniel Marchetti, DO; Aditi Satti, MD; Kartik Shenoy, MD; Robert M. Steiner, MD; Alex Swift, MD; Irene Swift, MD; Maria Elena Vega-Sanchez, MD

*University of Alabama, Birmingham, AL*: Mark Dransfield, MD; William Bailey, MD; Surya P. Bhatt, MD; Anand Iyer, MD; Hrudaya Nath, MD; J. Michael Wells, MD

*University of California, San Diego, CA*: Douglas Conrad, MD; Xavier Soler, MD, PhD; Andrew Yen, MD

*University of Iowa, Iowa City, IA*: Alejandro P. Comellas, MD; Karin F. Hoth, PhD; John Newell, Jr., MD; Brad Thompson, MD

*University of Michigan, Ann Arbor, MI*: MeiLan K. Han, MD MS; Ella Kazerooni, MD MS;

Wassim Labaki, MD MS; Craig Galban, PhD; Dharshan Vummidi, MD

*University of Minnesota, Minneapolis, MN*: Joanne Billings, MD; Abbie Begnaud, MD; Tadashi Allen, MD

*University of Pittsburgh, Pittsburgh, PA*: Frank Sciurba, MD; Jessica Bon, MD; Divay Chandra, MD, MSc; Joel Weissfeld, MD, MPH

*University of Texas Health, San Antonio, San Antonio, TX*: Antonio Anzueto, MD; Sandra Adams, MD; Diego Maselli-Caceres, MD; Mario E. Ruiz,
